# Structures of the R-type human Ca_v_2.3 channel reveal conformational crosstalk of the intracellular segments

**DOI:** 10.1038/s41467-022-35026-6

**Published:** 2022-11-30

**Authors:** Xia Yao, Yan Wang, Zhifei Wang, Xiao Fan, Di Wu, Jian Huang, Alexander Mueller, Sarah Gao, Miaohui Hu, Carol V. Robinson, Yong Yu, Shuai Gao, Nieng Yan

**Affiliations:** 1 Department of Molecular Biology, Princeton University, Princeton, NJ 08544 USA; 2 Department of Biological Sciences, St. John’s University, Queens, NY 11439 USA; 3 Physical and Theoretical Chemistry Laboratory, University of Oxford, Oxford, OX1 3QZ UK; 4 Kavli Institute for Nanoscience Discovery, University of Oxford, Oxford, OX1 3QU UK; 5 Present Address: Department of Radiology, Zhongnan Hospital of Wuhan University, School of Pharmaceutical Sciences, TaiKang Center for Life and Medical Sciences, Wuhan University, Wuhan, 430071 China

**Keywords:** Calcium channels, Cryoelectron microscopy, Membrane proteins, Permeation and transport

## Abstract

The R-type voltage-gated Ca^2+^ (Ca_v_) channels Ca_v_2.3, widely expressed in neuronal and neuroendocrine cells, represent potential drug targets for pain, seizures, epilepsy, and Parkinson’s disease. Despite their physiological importance, there have lacked selective small-molecule inhibitors targeting these channels. High-resolution structures may aid rational drug design. Here, we report the cryo-EM structure of human Ca_v_2.3 in complex with α2δ−1 and β3 subunits at an overall resolution of 3.1 Å. The structure is nearly identical to that of Ca_v_2.2, with VSD_II_ in the down state and the other three VSDs up. A phosphatidylinositol 4,5-bisphosphate (PIP2) molecule binds to the interface of VSD_II_ and the tightly closed pore domain. We also determined the cryo-EM structure of a Ca_v_2.3 mutant in which a Ca_v_2-unique cytosolic helix in repeat II (designated the CH2_II_ helix) is deleted. This mutant, named ΔCH2, still reserves a down VSD_II_, but PIP2 is invisible and the juxtamembrane region on the cytosolic side is barely discernible. Our structural and electrophysiological characterizations of the wild type and ΔCH2 Ca_v_2.3 show that the CH2_II_ helix stabilizes the inactivated conformation of the channel by tightening the cytosolic juxtamembrane segments, while CH2_II_ helix is not necessary for locking the down state of VSD_II_.

## Abstract (web summary)

Voltage-gated Ca2+ channel subtype Cav2.3 represents a potential drug target for neurological diseases. Here, authors report cryo-EM structures of Cav2.3 and its mutant to reveal the crosstalk of intracellular segments, which may facilitate future drug discovery.

## Introduction

Voltage-gated calcium (Ca_v_) channels permeate Ca^2+^ influx in response to the membrane depolarization. They regulate a broad range of physiological processes, such as muscle contraction, neurotransmitter release, hormone secretion, and cell death^1–4^. The 10 primary subtypes of mammalian Ca_v_ channels are divided into three subfamilies, Ca_v_1, Ca_v_2, and Ca_v_3, based on the phylogeny of the core α1 subunits. Ca_v_1 and Ca_v_3 channels are also known as the L-type and T-type channels, respectively, for their distinct electrophysiological properties. The three Ca_v_2 members, known as the P/Q-type for Ca_v_2.1, the N-type for Ca_v_2.2, and the R-type for Ca_v_2.3, play an important role in signal transduction in the central and peripheral nervous systems^1–5^.

A systematic structural analysis of Ca_v_ channels will not only reveal the molecular basis for their working principles, but also facilitate drug discovery targeting various Ca_v_ channelopathies^4,6,7^. Benefiting from the resolution revolution of single-particle cryo-electron microscopy (cryo-EM), we have solved the high-resolution structures of representative members from each Ca_v_ subfamily, Ca_v_1.1, Ca_v_1.3, Ca_v_2.2, and Ca_v_3.1 over the years since 2015^8–12^. These structures reveal the common architecture of Ca_v_ channels. The transmembrane regions of the α1 subunits all share the canonical voltage-gated ion channel fold, wherein four homologous repeats (designated I-IV), each containing six transmembrane helices (S1-S6), are interwoven in a domain-swapped manner^13,14^. The four sets of S5 and S6 segments enclose the central pore domain (PD), which is responsible for the selective Ca^2+^ permeation. The S1-S4 segments in each repeat constitute the voltage sensing domains (VSD) that undergo conformational shifts in response to membrane potential changes, transmitting the electric signals to pore gating^15–18^.

More rewarding are structural discoveries of the subtype-specific features. Of particular note, an endogenous phosphatidylinositol 4,5-bisphosphate (PIP2) molecule is seen to attach to the interface of VSD_II_ and the PD on the inner membrane leaflet of human Ca_v_2.2^11,19^. In addition, the exceptionally long S6_II_ segment is followed by two consecutive helices, designated CH1_II_ and CH2_II_, which fold back toward the membrane. A Trp residue that marks the amino terminus of CH2_II_ appears to play a critical role to secure the closed state of the intracellular gate^11,19^. To investigate whether these structural features are unique to Ca_v_2.2 or conserved in the Ca_v_2 subfamily, we sought to solve the structures of human Ca_v_2.1 and Ca_v_2.3. As of now, we have not been able to produce suitable samples of Ca_v_2.1 for cryo-EM analysis. Our present study focuses on Ca_v_2.3.

Ca_v_2.3 channels, a ternary complex comprising the extracellular α2δ and the cytosolic β subunits in addition to the α1 subunit, are strongly expressed in the cortex, hippocampus, striatum, amygdala, and interpeduncular nucleus^20^. Six different splice variants of the α1 subunits, Ca_v_2.3a-f, have been identified in various mammalian species^21^. Dysfunction of Ca_v_2.3 is the major cause of developmental and epileptic encephalopathy 69 (DEE69), a severe encephalopathic disorder characterized by refractory seizures and neurodevelopmental impairment^22^. Ca_v_2.3-deficient mice display reduced seizure activity, altered pain response, and protection from Parkinson’s disease-related neurodegeneration^23–25^. Therefore, Ca_v_2.3 channels represent potential drug targets for managing epileptic seizures and neurological disorders^26^.

Unlike the P/Q-type and the N-type Ca_v_ channels, the Ca_v_2.3 channels display low sensitivities to ω-conotoxins, a group of neurotoxic peptides that have been used as tools to explore the physiological functions of Ca_v_2 channels^27,28^. Ca_v_2.3 channels are subject to selective inhibition by SNX-482, a 41-aa peptide toxin from the tarantula *Hysterocrates gigas*, which exhibits an antinociceptive activity^26^. Furthermore, the Ca_v_2.3 channels show a more rapid inactivation and slower recovery from inactivation, which contributes to their specific functional roles in neurons^29^.

Here, we report the structural analysis of the human Ca_v_2.3 complex. The high-resolution structure provides an accurate template for drug discovery. To investigate the role of the CH2_II_ helix, we determined the cryo-EM structures of a CH2_II_-truncated mutant (named as ΔCH2) in addition to the full-length channel. Our studies show that CH2_II_ stabilizes the inactivated conformation of the channel by tightening the juxtamembrane region on the cytosolic side.

## Results

### Nearly identical structure of full-length Ca_v_2.3 with Ca_v_2.2

The full-length human Ca_v_2.3 ternary complex consisting of α1, α2δ−1, and β3 was recombinantly co-expressed, purified, and analyzed with cryo-EM using a protocol nearly identical to that for Ca_v_2.2^11^. The only modification is the addition of 220 μM SNX-482, that was reported to specifically inhibit Ca_v_2.3 by binding to VSDs III and IV^26,30^. However, there was no discernible density for the peptide toxin throughout data processing. It is possible that the purification condition, which disrupts the membrane potential, is incompatible with SNX-482 binding, as depolarization was reported to reverse toxin association^30^.

**Fig. 1:**
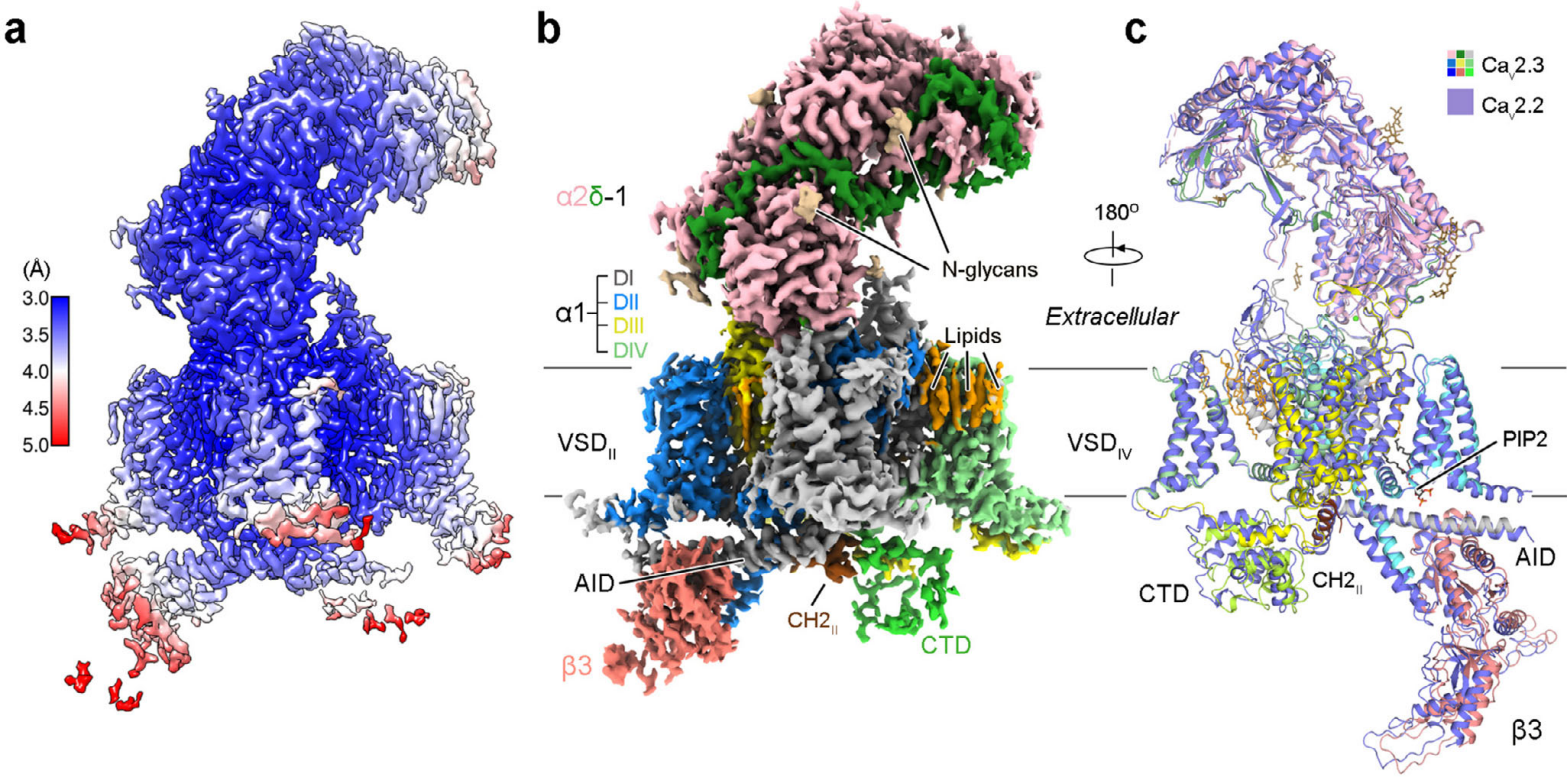
Cryo-EM structure of the human Ca_v_2.3 complex. **a** Heat map for the local resolutions of the Ca_v_2.3 EM reconstruction. The map, calculated in RELION, was generated in ChimeraX. **b** 3D EM map of human Ca_v_2.3 complex comprising the extracellular α2δ−1 subunit (light pink for α2, and green for δ), the transmembrane α1 subunit (domain-colored), and the cytosolic β3 subunit (salmon). The cytosolic helix of repeat II, designated as CH2_II_, is colored chocolate. Sugars and lipids are colored wheat and orange, respectively. Also labeled are the α1-interacting domain (AID) and the carboxy-terminal domain (CTD). The same color scheme is applied to the structure of full-length Ca_v_2.3 in the manuscript unless otherwise indicated. **c** Nearly identical structures of Ca_v_2.2 and Ca_v_2.3. The structures of Ca_v_2.2 (purple, PDB code 7MIY) and Ca_v_2.3 (domain-colored) can be superimposed with the root-mean-square deviation (RMSD) of 0.81 Å for 1905 Cα atoms in the α1 and α2δ−1 subunits. Due to the low resolution, the β3 structure from the Ca_v_2.2 complex was docked as a rigid body in the map of Ca_v_2.3. PIP2 (black sticks) binds at the interface of VSD_II_ and the PD of human Ca_v_2.3 in the inner membrane leaflet.

A 3D EM reconstruction for the Ca_v_2.3 ternary complex was determined at an average resolution of 3.1 Å (Fig. 1a, b, Supplementary Figs. 1, 2 and Supplementary Table 1). As seen in other Ca_v_ channel complexes^9,11^, α1 and α2δ−1 subunits are well resolved, while β3 is of lower resolution. The structure of β3 from the Ca_v_2.2 complex (PDB: 7MIY) was docked as a rigid body.

The overall structure of Ca_v_2.3 is nearly identical to that of apo-Ca_v_2.2, with the root-mean-square deviation (RMSD) of 0.81 Å for 1905 Cα atoms in the α1 and α2δ−1 subunits (Fig. 1c). Briefly, the α1 subunit is also featured with a down VSD_II_, while the other three VSDs are in the typical depolarized or up conformations (Supplementary Fig. 3). The closed intracellular gate of PD is secured by CH2_II_. And a PIP2 molecule bound to the same position is also clearly resolved (Fig. 1c and Supplementary Fig. 2b). Five PIP2 species, 34:1, 36:2, 36:1, 38:3, and 38:2, were detected in the lipidomic analysis of purified channel complex (Supplementary Fig. 2c). These observations suggest that the structural and functional roles of CH2_II_ and PIP2 may be conserved in the N- and R-type Ca_v_ channels. The shared structural features of Ca_v_2.2 have been thoroughly depicted in our previous report^11^. Here we will avoid redundant structural illustrations, but focus on CH2_II_ for detailed analysis.

### CH2_II_ stabilizes an inactivated state of the channel

**Fig. 2:**
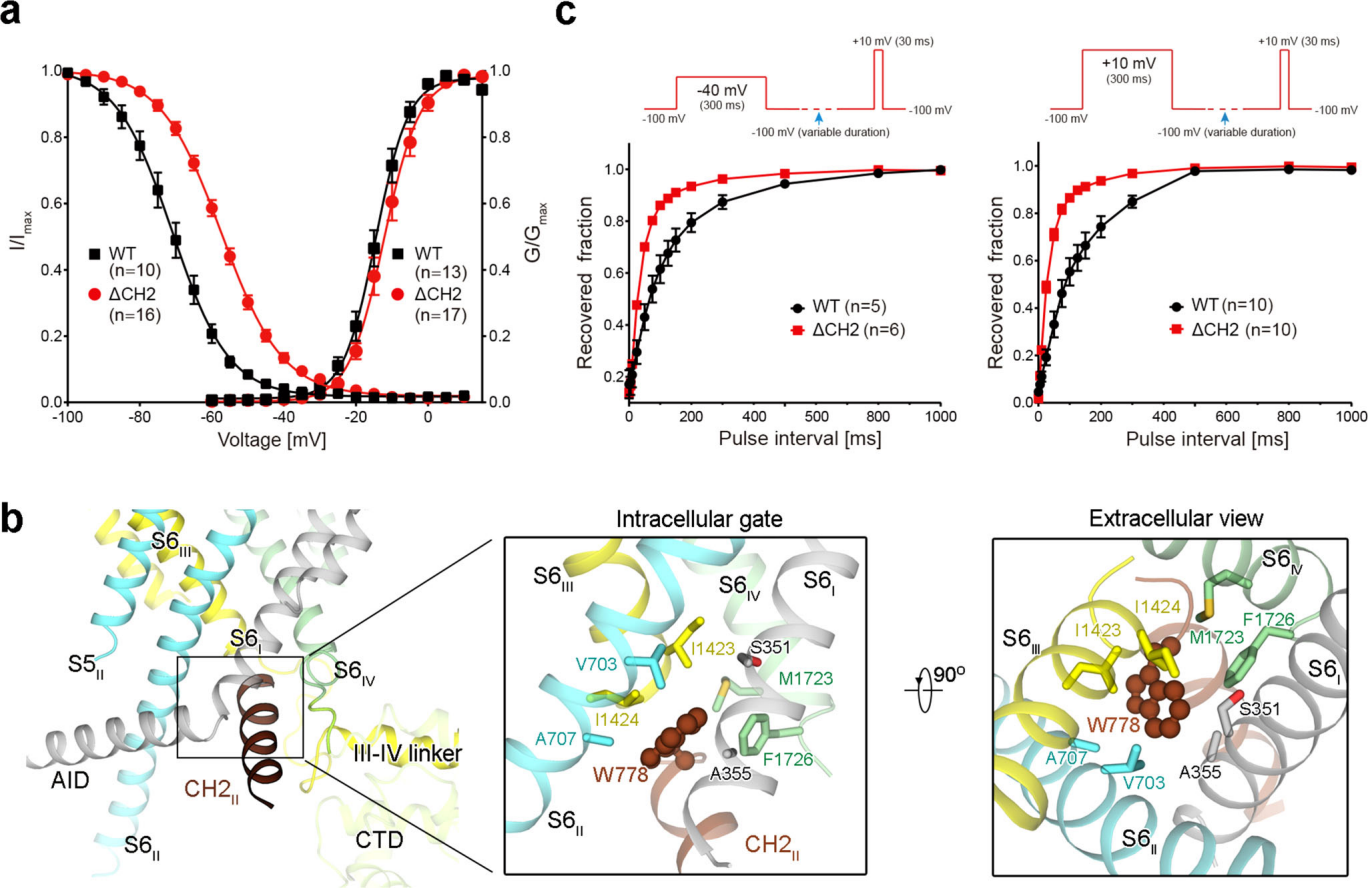
CH2_II_ stabilizes an inactivated state of the channel. **a** Deletion of the entire CH2_II_ segment (mutant named as ΔCH2) did not affect channel activation, but led to a pronounced right shift in the steady-state inactivation. Half-maximal activation: V_1/2_ (WT) = −14.7 ± 0.3 mV, V_1/2_ (ΔCH2) = −12.3 ± 0.2 mV; Half-maximal inactivation: V_1/2_ (WT) = −70.9 ± 0.1 mV, V_1/2_ (ΔCH2) = −57.3 ± 0.1 mV. All data are presented as mean ± SEM. The number of tested cells is labeled in parentheses. Source data are provided as a Source Data file. **b** CH2_II_ (colored chocolate) is positioned right beneath the intracellular gate and surrounded by multiple intracellular segments. Inset: CH2_II_ tightens the closed intracellular gate. Trp778 (chocolate sphere) at the beginning of CH2_II_ helix interacts with gating residues (domain-colored sticks) on the S6 tetrahelical bundle. **c** ΔCH2 mutant recovers from inactivation much faster than WT channel. The electrophysiological protocols are shown above the curves. After treating with pre-pulses at −40 mV (left) or +10 mV (right) for 300 ms to induce inactivation, −100 mV recovery voltage was applied for variable times (0-1000 ms), which is then followed by a 30-ms test pulse at +10 mV. For pre-pulse at −40 mV, the fitted time constants (τ) are 90.3 ± 5.4 ms and 33.9 ± 1.3 ms for WT and ΔCH2, respectively. For pre-pulse at +10 mV, τ = 137.4 ± 4.0 ms for WT and 40.0 ± 2.0 ms for ΔCH2. Source data are provided as a Source Data file.

Ca_v_2.3 has a similar voltage dependence for activation and steady-state inactivation to Ca_v_2.2, with V_1/2_ measured at −14.7 ± 0.3 mV and −70.9 ± 0.1 mV, respectively (Fig. 2a and Supplementary Figs. 4a–c). When the holding potential was set at −100 mV, more than 90% of the Ca_v_2.3 channels activated at 0 mV (Fig. 2a). But when the holding potential was at −40 mV or more depolarized, most channels were trapped in the inactivated state (Fig. 2a). As the recombinant channels were expressed in HEK293F cells and purified at 0 mV, the purified channels are likely trapped in an inactivated state. As briefly mentioned in the introduction, a Trp residue at the beginning of CH2_II_ serves as an organizing center for intracellular gating residues. In analogy, it is like a bolt that tightens the S6 tetrahelical bundle at the intracellular gate (Fig. 2b).

Supporting the structural analysis, deletion of the entire CH2_II_ helix (residues 773-791, the resulting mutant named ΔCH2) had no impact on channel activation, but shifted the V_1/2_ for the steady-state inactivation from −70.9 ± 0.1 mV to −57.3 ± 0.1 mV (Fig. 2a and Supplementary Fig. 4c). We then measured the rates of recovery for WT and ΔCH2 treated at different pre-pulses. For these experiments, pre-pulses of −40 mV or 10 mV were respectively applied for 300 ms to induce inactivation. After holding the cells at the hyperpolarization of −100 mV for variable durations, the channel opening at 10 mV was recorded (Fig. 2c and Supplementary Fig. 4d). Supporting the role of CH2_II_ to secure the inactivated state, ΔCH2 mutant recovered faster than WT channel under both settings (Fig. 2c). For recovery from the pre-holding at −40 mV, the fitted time constants (τ) of WT and ΔCH2 channels are 90.3 ± 5.4 ms and 33.9 ± 1.3 ms, respectively (Fig. 2c, left). When pre-holding was set at 10 mV, ΔCH2 also accelerated the recovery with τ shortened from 137.4 ± 4.0 ms to 40.0 ± 2.0 ms (Fig. 2c, right). The electrophysiological characterizations consolidate the structural analysis that the CH2_II_ helix stabilizes the inactivated conformation of Ca_v_2.3.

As CH2_II_ is unique to Ca_v_2 channels and only Ca_v_2.2 and Ca_v_2.3 show a down VSD among all resolved Ca_v_ structures, we next examined if ΔCH2 would display a similar conformation to the structures of Ca_v_1.1 and Ca_v_1.3, in which all four VSDs are up^9,12^.

### The juxtamembrane domains are less ordered in ΔCH2

The protein expression level and solution behavior of ΔCH2 are similar to that of the WT channel complex. Following our standard protocol, the structure of ΔCH2 was determined at an overall resolution of 3.1 Å out of 68,109 particles (Supplementary Fig. 5). Despite a similar nominal resolution for the overall structure to that of the WT channel, several functional units were only poorly resolved in ΔCH2.

**Fig. 3:**
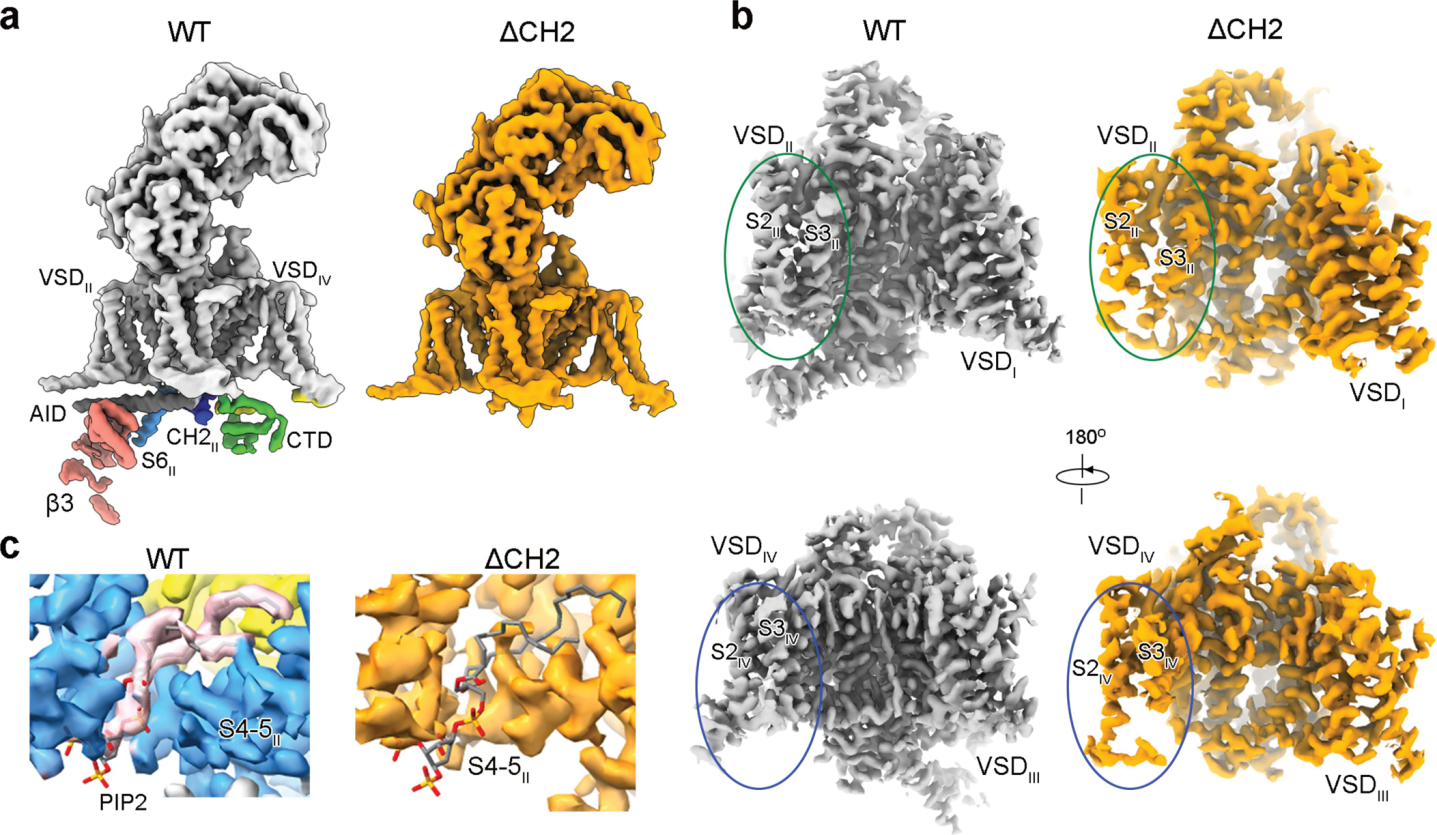
The juxtamembrane domains are less ordered in the absence of CH2_II_. **a** Almost all the intracellular segments, including the β3 subunit, the AID, the cytosolic fragment of S6_II_, and the CTD are barely discernible in the map of ΔCH2. The unresolved segments are highlighted in the map of full-length channel (WT) with the same color scheme as in Fig. 1b. For visual clarity, both maps are low pass-filtered to 6 Å. **b** VSD_II_ (top) and VSD_IV_ (bottom) display discontinuous densities for the S2 and S3 segments in the absence of the CH2_II_ helix. **c** PIP2 is not resolved in ΔCH2 map. The density for PIP2, colored pink in the WT map, is missing in the map of ΔCH2.

In the absence of CH2_II_, the segments on the intracellular border of the membrane and in the cytosol, including the α1-interacting domain (AID), which is the elongated helix succeeding S6_I_, the cytosolic fragment of S6_II_, the III-IV linker and the carboxy-terminal domain (CTD), are barely discernible in the final map (Fig. 3a). The entire β3 subunit is completely invisible. Within the transmembrane region, VSD_II_ and VSD_IV_ display lower local resolutions, especially for their S2 and S3 segments (Fig. 3b). These observations support that CH2_II_ stabilizes the intracellular side of the juxtamembrane region of the channel.

Of particular note, there is no density for PIP2 in the 3D reconstruction of ΔCH2 (Fig. 3c). To distinguish if the invisibility of PIP2 is due to loss of binding or poor resolution associated with local structural flexibility, we characterized the presence of PIP2 in WT and ΔCH2 using lipidomic analysis. The abundance of PIP2 indeed dropped in ΔCH2 by about a half (Supplementary Fig. 6).

### CH2_II_ couples PD gating

**Fig. 4:**
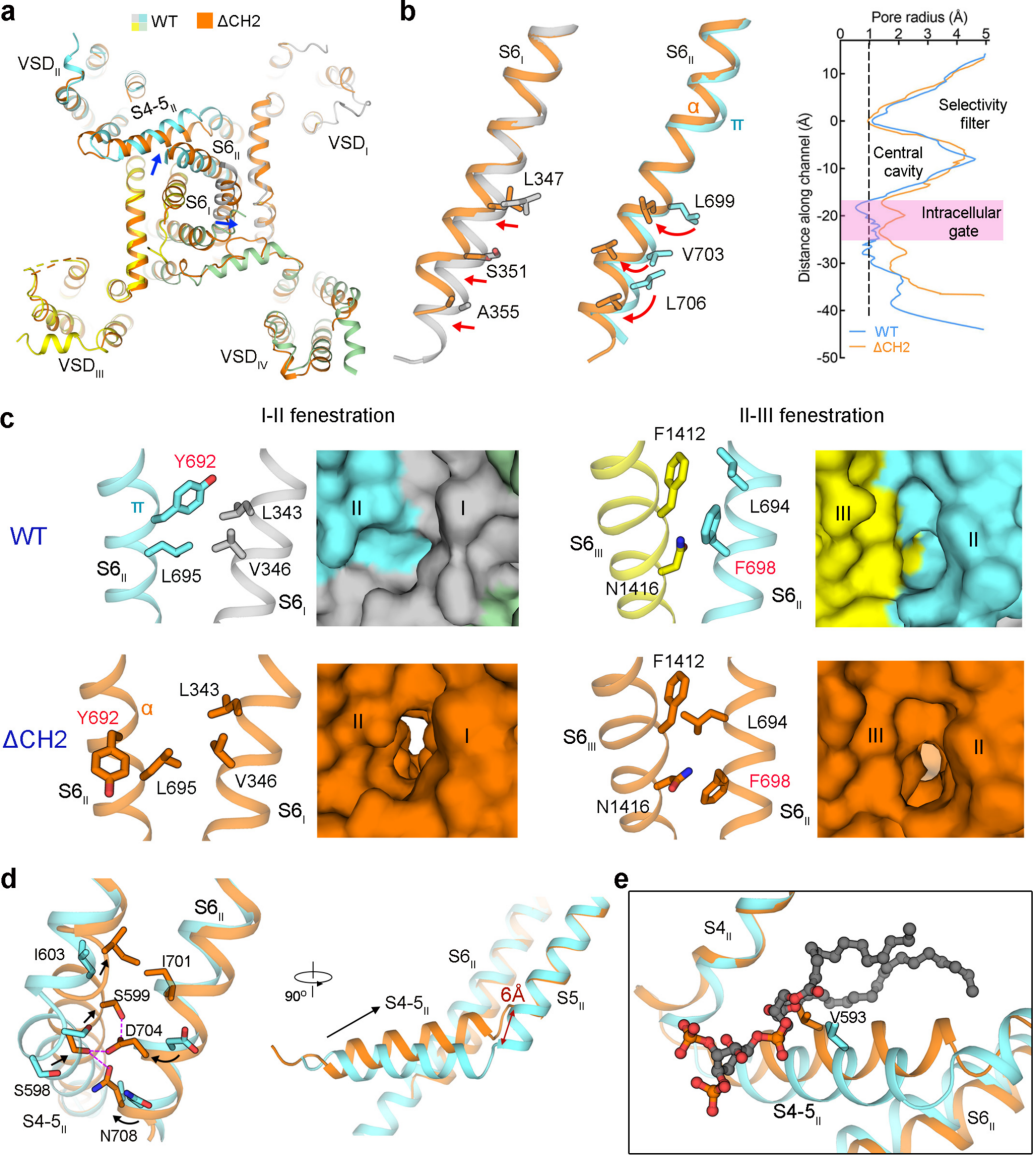
Conformational changes of the PD upon CH2 deletion. **a** In the superimposed α1 structures of WT and ΔCH2 Ca_v_2.3, S6_I_, S6_II_ and S4-5_II_ segments exhibit evident conformational changes. Blue arrows indicate the structural shifts from WT to ΔCH2. **b** The gating residues on S6_I_ displace outwards (left), while those on S6_II_ undergo an axial rotation (middle). The structural shifts of gating residues from WT to ΔCH2 are indicated by red arrows. A secondary structure transition from π → α occurs in S6_II_. Right: In the absence of CH2_II_, the intracellular gate becomes loosely closed. The pore radii are calculated by HOLE^53^. Source data are provided as a Source Data file. **c** Opening of the I-II (left) and II-III (right) fenestration sites upon the axial rotation of S6_II_ that turns Tyr692 and Phe698 (labeled red) away in ΔCH2. **d** Rotations of Asp704 and Asn708 on S6_II_ induce the upward motion of S4-5_II_ and its joint with S5_II_. Purple dashes indicate the H-bonds between Ser588 and Ser589 on S4-5_II_ with Asp704 and Asn708 on S6_II_. Structural shifts of the interacting residues are indicated by black arrows. Right: The hinge between S4-5 and S5_II_ moves toward the membrane by a displacement of ~ 6 Å. **e** The local shift of S4-5_II_ segment disfavors PIP2 binding. The upward movement would lead to potential clash with PIP2 binding.

Next, we compared the structures of ΔCH2 and WT Ca_v_2.3 for detailed analysis. A total of 1939 out of 3416 side chains were assigned for α2δ−1 and the extracellular and transmembrane region of α1 in ΔCH2 (Supplementary Fig. 7 and Supplementary Table 1). For the α1 segments with lower resolutions, such as the S3 segments in VSD_II_ and VSD_IV_, poly-Ala were assigned. The overall structures of the α1 subunit of ΔCH2 and WT Ca_v_2.3 can be superimposed with a RMSD of 0.57 Å over 775 Cα atoms (Fig. 4a). We had expected that VSD_II_ would exhibit an “up” conformation; however, it is clear that VSD_II_ in ΔCH2 remains down, identical to that in the WT channel (Supplementary Fig. 8a). In contrast, the ensuing S4-5_II_ and the PD exhibit evident structural deviations (Fig. 4a and Supplementary Fig. 8b).

Both S6_I_ and S6_II_ slightly move outwardly, resulting in a loosened but still closed intracellular gate (Fig. 4b). Accompanying the rotation of S6_I_ and S6_II_, the previously sealed I-II and II-III side walls of the PD now both have fenestrations in ΔCH2 (Fig. 4c). Therefore, the PD in the WT Ca_v_2.3 is in a tightly closed state, whereas that in ΔCH2 is loosely closed, as seen in the structures of ligand-free Ca_v_1.1, Ca_v_1.3,and Ca_v_3.1^9,10,12^. Consistent with previous structural observations in Ca_v_ and Na_v_ channels^31^, S6_II_ has one π-helical turn in the tightly closed WT channel, which transforms to the α conformer in the ΔCH2 structure (Fig. 4b, c).

Rotation of S6_II_ places two polar residues, Asp704 and Asn708, to face S5_II_ and S4-5_II_. In the structure of ΔCH2, the S4-5_II_ segment and its hinge with S5_II_ undergo the most pronounced structural shift; the hinge rises towards the membrane by approximately 6 Å. In this conformation, Asp704 on S6_II_ can form hydrogen bonds (H-bonds) with Ser598 and Ser599, and Asn708 is also H-bonded to Ser598 (Fig. 4d). A lifted hinge between S4-5_II_ and S5_II_ together with an unchanged VSD_II_ result in a sharpened angle between S4_II_ and S4-5_II_, which is no longer compatible with PIP2 binding. This conformational change thus explains the lack of PIP2 density in the map of ΔCH2 (Fig. 4d, e and Supplementary Fig. 8b).

### Distinct conformations of AID in ΔCH2

**Fig. 5:**
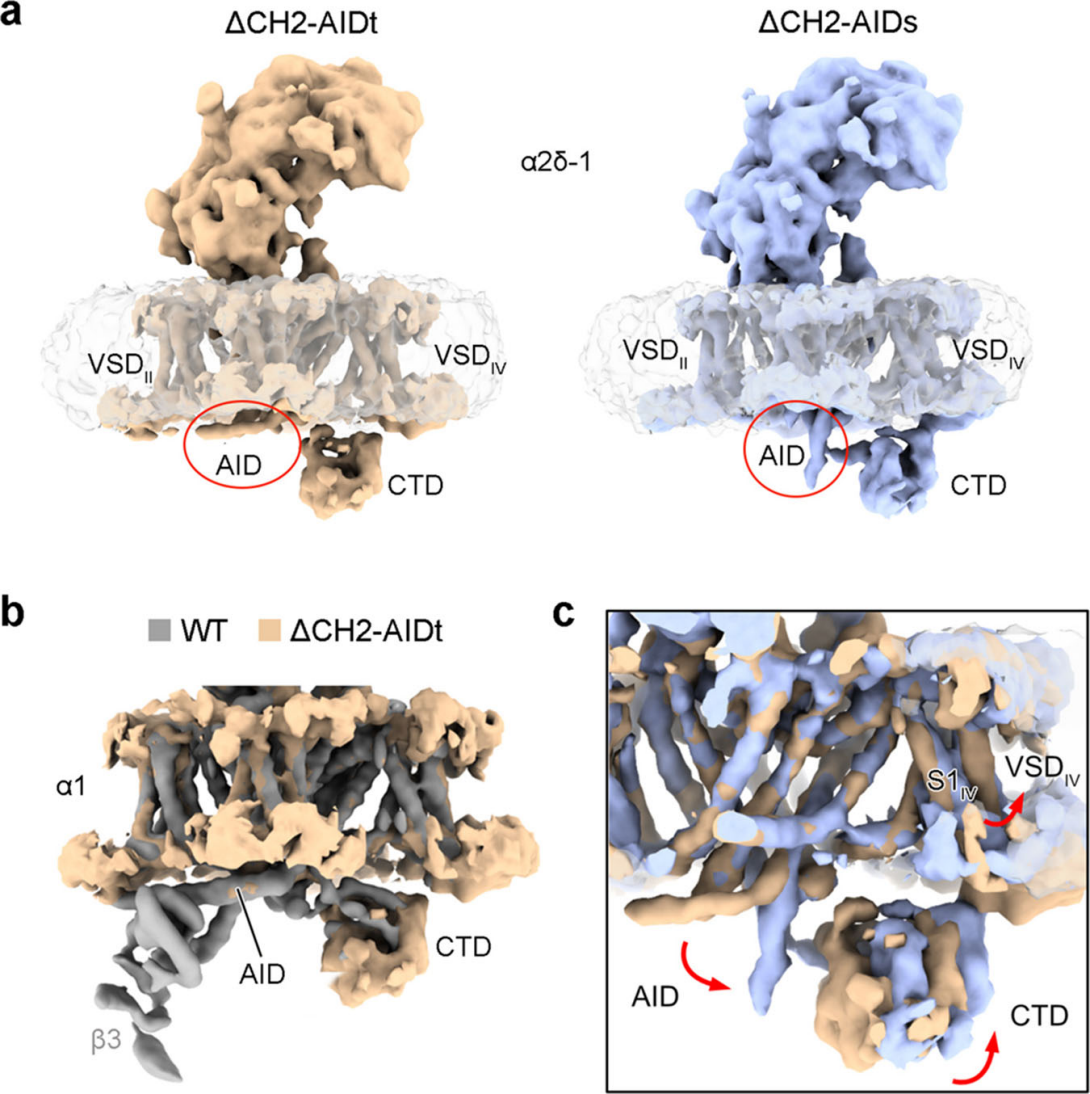
3D variability analysis (3DVA) of ΔCH2 mutant Ca_v_2.3. **a** Two extreme trajectories from 3DVA reveal a “transverse” AID (1^st^ frame, designated as ΔCH2-AIDt) versus a “straight” AID (10^th^ frame, designated as ΔCH2-AIDs). These two reconstructions are low pass-filtered to 7 Å. **b** The α1 subunit in ΔCH2-AIDt (wheat) and WT Ca_v_2.3 (gray) can be well aligned. **c** Accompanying the straightening of AID, CTD and VSD_IV_ also undergo a concerted swing. Red arrows indicate the structural shifts from ΔCH2-AIDt (wheat) to ΔCH2-AIDs (light blue). Also referred to Supplementary Movie 1 for the conformational shifts.

Although the intracellular region is invisible in the final high-resolution map, there are still densities in the low pass-filtered map. We attempted to probe the structural heterogeneity of ΔCH2 using 3D variability analysis (3DVA) in cryoSPARC^32^. Potential motion trajectories were analyzed in multi-dimensional conformational space, in which one mode shows a significant dynamic motion of the AID, CTD, and VSD_IV_ (Fig. 5, Supplementary Fig. 5d and Supplementary Movie 1).

Two extreme trajectories, the 1^st^ (designated as ΔCH2-AIDt) and the 10^th^ (ΔCH2-AIDs) frames of the reconstructed volume series, together with the full transition along the volume series reveal a marked conformational shift of the AID from a WT-like “transverse” conformation to a “straight” one (Fig. 5a, b and Supplementary Movie 1). Meanwhile, the CTD undergoes a concerted swing accompanying the straightening of the AID (Fig. 5c and Supplementary Movie 1). VSD_IV_, particularly its S1_IV_ segment that is directly connected to the III-IV linker that binds to the CTD, moves along the same direction but to a smaller degree than the CTD (Supplementary Movie 1).

## Discussion

Our present study set out to address the following questions. 1) Is the Ca_v_2-unique CH2_II_ helix the determinant for the down state of VSD_II_ in the cryo-EM structure? 2) What are the functional and structural roles of the CH2_II_ helix? Our structural and electrophysiological characterizations of human Ca_v_2.3 confirm the role of the CH2_II_ helix in stabilizing the inactivated conformation of the channel. In the structure of CH2_II_-deleted Ca_v_2.3, the PD is loosely closed, but VSD_II_ remains down. The overall conformation is no longer compatible with PIP2 binding. Therefore, the structure of ΔCH2 demonstrates that the CH2_II_ helix is not necessary for the down state of VSD_II_. Sequence analysis cannot offer an immediate clue to the determinant for the down state of VSD_II_. Future characterizations, such as engineering of Ca_v_1 and Ca_v_2 chimera followed by the systematic mutagenesis, may unveil the determinants for the conformational switch of VSD_II_.

An unexpected discovery is the conformational flexibility of the AID. In all the reported structures of Ca_v_1.1, Ca_v_1.3, and Ca_v_2.2^8,9,11,12,19,33,34^, AID is a transverse helix lying on the intracellular surface of the membrane, connected to S6_I_ through a short turn. 3DVA of the low pass-filtered map of ΔCH2 reveals a potentially straightened conformation of the AID as a natural extension of S6_I_ into the cytosol. When the maps were further low pass-filtered to ~15 Å, additional densities on the cytosolic side were shown (Supplementary Fig. 9). In reference to the low pass-filtered map for WT Ca_v_2.3, this density likely belongs to the β3 subunit, which is invisible in the high-resolution map (Fig. 3a and Supplementary Fig. 9). The position and contour of the density in ΔCH2-AIDt are similar to that in the WT map (Supplementary Fig. 9, upper row). In the map of ΔCH2-AIDs, however, the density appears to undergo a large degree of rotation (Supplementary Fig. 9, lower row). If this density indeed corresponds to β3 that binds to AID in both bent and straightened conformation, the shift of the density is consistent with the shift of AID. But as the density can only be seen in the low pass-filtered maps analyzed with 3DVA, we cannot rule out the possibility that this density may not belong to β3.

In light of the potential conformational diversity of AID and β3 in ΔCH2, we re-examined the maps of WT Ca_v_1.1, Ca_v_2.2, and Ca_v_2.3 (EMDB codes: EMD-22426, EMD-23868, and EMD-28529, respectively). In the presence of CH2_II_, the β3 subunit is sandwiched between the transverse AID and the elongated S6_II_ in Ca_v_2.2 and Ca_v_2.3. There is no additional conformation for the AID. In Ca_v_1.1, which does not possess a CH2_II_ helix, AID and the bound β1 do show certain conformational heterogeneities, but AID remains to be bent^9^. Of note, there is one major difference between WT Ca_v_1.1 and Ca_v_2.3-ΔCH2. VSD_II_, which interacts with the bent AID, is up in Ca_v_1.1 and down in Ca_v_2.3-ΔCH2. Considering that the β1 subunit is critical for the coupling between Ca_v_1.1 and the downstream RyR1 in skeletal muscle^35–37^, the dramatic conformational shift of AID indicated by the 3DVA analysis immediately raised the question of whether AID is straightened in a resting state Ca_v_1.1 channel, wherein VSD_II_ is down, a speculation to be tested in the future.

Ca_v_2.3 channels represent potential targets for the treatment of various neurological diseases^22,38^. However, there have lacked effective and selective Ca_v_2.3 modulators as of today. Our structures provide an accurate molecular template for mapping disease mutations (Supplementary Fig. 10) and may facilitate the structure-based drug design^22^. Our structure-based discoveries serve as a framework to probe the electromechanical coupling of Ca_v_ channels and shed light on the mechanistic investigation of the excitation-contraction coupling of skeletal muscles that involve Ca_v_1.1 and β1.

## Methods

### Transient expression of human Ca_v_2.3 in HEK293F cells

Codon-optimized cDNAs of *CACNA1E* for full-length Ca_v_2.3 α1 (2,313 residues, Uniprot Q15878-1), *CACNA2D1* for α2δ−1 (1,103 residues, Uniprot P54289-1) and *CACNB3* for β3 (484 residues, Uniprot P54284-1) were synthesized (BGI Geneland Scientific). For CH2-deleted Ca_v_2.3 (ΔCH2), residues 773-791 of the α1 subunit were deleted with standard two-step PCR. All the subunits were cloned into the pCAG vector, with an amino-terminal Flag-tag and a carboxy-terminal His_10_-tag at the α1 and β3 subunits. For western blotting to examine the stoichiometry of the α1 and β3 subunits, β3 subunit was cloned into the pCAG vector with amino-terminal twin strep-tag. HEK293F suspension cells (Thermo Fisher Scientific, R79007) were cultured in Freestyle 293 medium (Thermo Fisher Scientific) at 37 °C, supplied with 5% CO_2_ under 60% humidity. When cell density reached 1.5-2.0 × 10^6^ cells per mL, a mixture of expression plasmids including 0.75 mg α1, 0.6 mg α2δ−1 and 0.5 mg β3, and 3 mg polyethylenimine (Polysciences) were added into the cell culture for transient expression of human Ca_v_2.3 complex.

### Protein purification of human Ca_v_2.3

For one batch of protein purification, 8 L of HEK293F cells were collected approximately 72 h after transfection by centrifugation at 3600 g for 10 min and resuspended in the lysis buffer containing 25 mM HEPES (pH 7.4), 150 mM NaCl, 2 mM CaCl_2_ and the protease inhibitor cocktail containing 2.6 μg mL^−1^ aprotinin (VWR Life Science) and 1.4 μg mL^−1^ pepstatin (VWR Life Science). The suspension was supplemented with glycol-diosgenin (GDN, Anatrace) to a final concentration of 1% (w/v), n-dodecyl-β-D-maltopyranoside (DDM, Anatrace) to 0.2% (w/v), and cholesteryl hemisuccinate Tris salt (CHS, Anatrace) to 0.04% (w/v). After incubation at 4 °C overnight, the mixture was centrifuged at 35,000 g for 30 min, and the supernatant was applied to anti-Flag M2 affinity resin (Sigma) for affinity purification. The resin was rinsed with wash buffer (buffer W) containing 25 mM HEPES (pH 7.4), 150 mM NaCl, 2 mM CaCl_2_, and 0.01% GDN and eluted with buffer W supplemented with 0.2 mg mL^−1^ Flag peptide (synthesized by GenScript). The eluent was then concentrated using a 100-kDa molecular weight cut-off Amicon filter unit (Millipore) and further purified through size-exclusion chromatography (Superose 6 10/300 GL, GE Healthcare) that was pre-equilibrated in buffer W. The peak fractions were pooled and concentrated to a final concentration of about 20 mg mL^−1^ with α2δ−1 in excess.

For structural determination of Ca_v_2.3 in the complex with peptide toxin, purchased SNX-482 (Alomone labs) was only added to the concentrated wild-type protein solution at a final concentration of 220 μM. The mixture was incubated at 4 °C for 30 min before making cryo-grids.

For western blotting, 1.5 L of HEK293F cells expressing the wild-type or ΔCH2 mutant Ca_v_2.3 proteins were purified following the same procedure. The eluents after anti-Flag resin were separated by SDS-PAGE and transfected onto PVDF membrane (Millipore). The membranes were blocked by 5% (w/v) nonfat milk (Bio-Rad) in TBST buffer containing 25 mM Tris (pH 8.0), 150 mM NaCl, and 0.05% (w/v) Tween-20. The membranes were incubated with primary monoclonal anti-Flag® M2 antibody (1:3000 dilution, Sigma-Aldrich) against the α1 subunit or Strep Tag II monoclonal antibody (1:2500 dilution, Invitrogen, clone 1810CT579.47.56.10) against the β3 subunit, and IRDye® 800CW goat anti-mouse IgG secondary antibody (1:4000 dilution, Li-COR). The membranes were exposed by Odyssey® CLx imaging system (LI-COR 9140).

### Cryo-EM sample preparation and data collection

Aliquots of 3.5 μl concentrated WT or ΔCH2 Ca_v_2.3 proteins were loaded onto glow-discharged holey carbon grids (Quantifoil Cu/Au R1.2/1.3, 300 mesh), which were blotted for 6 s and plunge-frozen in liquid ethane cooled by liquid nitrogen using a Vitrobot Mark IV (Thermo Fisher) at 8 °C with 100% humidity. Grids were transferred to a Titan Krios electron microscope (Thermo Fisher) operating at 300 kV and equipped with a Gatan Gif Quantum energy filter (slit width 20 eV) and spherical aberration (Cs) image corrector. Micrographs were recorded using a K2 Summit counting camera (Gatan) in super-resolution mode with a nominal magnification of 105,000×, resulting in a super-resolution pixel size of 0.557 Å. Each stack of 32 frames was exposed for 5.6 s, with an exposure time of 0.175 s per frame. The total dose for each stack was about 50 e^−^ per Å^2^. The dose rate is 10.6 e^-^/ pixel/s. SerialEM was used for fully automated data collection^39^. All 32 frames in each stack were aligned, summed, and dose-weighted using MotionCorr2^40^ and twofold-binned to a pixel size of 1.114 Å per pixel. The defocus values were set from −1.9 to −2.1 μm and estimated by Gctf^41^.

### Image processing

For WT Ca_v_2.3, the images were collected in three batches. Although the last two batches of samples were added SNX-482, no density for the drug was observed. A total of 7,160 (1^st^: 2,568; 2^nd^: 2,245; 3^rd^: 2,347) cryo-EM micrographs were collected and 1,664,705/1,962,632/2,117,770 particles were auto-picked by RELION-3.0^42^. Particle picking was performed using 2D classes of human Ca_v_2.2 (EMD-23868) in the side and tilted views as reference. All subsequent 2D and 3D classification and refinement were performed with RELION-3.0. One round of reference-free 2D classification was performed to remove ice spots, contaminants, and aggregates, yielding 1,415,239/1,692,632/2,072,104 particles. The particles were then processed with the global search 3D multi-reference classification with K = 4 using bin2 particles. The EM map of human Ca_v_2.2 (EMD-23868)^11^, low pass-filtered to 20 Å, was used as an initial good reference. The output of 1^st^ and 2^nd^ datasets were applied to the local angular search 3D classification with four classes. The 3^rd^ dataset was performed skip align 3D classification. A total of 221,390/345,032/50,314 particles were selected by combining the good classes. The particles were then re-extracted using a box size of 280 pixels and pixel size of 1.114 Å. These particles yielded reconstructions at 3.7/4.5/3.7 Å after 3D auto-refinement with an adapted mask. Skip align 3D classification for 1^st^ and 2^nd^ datasets using bin1 particles and Bayesian polishing for all datasets resulted in reconstructions at 3.2/3.6/3.5 Å from 47,971/64,770/50,314 particles. Skip align 3D classification for the merged particles from three datasets afforded the final reconstruction at 3.1 Å out of 118,244 particles.

For ΔCH2, A total of 6,160 (1^st^: 1,930; 2^nd^: 2,563; 3^rd^: 1,667) cryo-EM micrographs were collected and 1,530,824/1,696,892/1,236,150 particles were auto-picked. One round of reference-free 2D classification yielded 1,500,417/1,535,373/1,123,979 particles. The particles were then processed with a global search 3D multi-reference classification with K = 4 using bin2 particles. The particles of 1^st^ dataset were then applied to skip align 3D classification, and the output of 2^nd^ and 3^rd^ datasets were then processed with local angular search 3D classification. A total of 55,655/329,382/184,237 particles were selected by combining the good classes. Bin1 particles yielded reconstructions at 4.4/4.2/4.3 Å after 3D auto-refinement with an adapted mask. Skip align 3D classification for 2^nd^ dataset only and Bayesian polishing for all datasets resulted in reconstructions at 4.2/3.2/3.6 Å from 55,655/38,415/30,126 particles. Skip align 3D classification for the merged particles yielded the reconstruction at 3.1 Å out of 68,109 particles. For WT and ΔCH2, > 97% of junk particles and low-resolution particles were thrown out during data processing.

All 2D classification, 3D classification, and 3D auto-refinement were performed with RELION 3.0. Resolutions were estimated using the gold-standard Fourier shell correlation 0.143 criterion with high-resolution noise substitution^43,44^.

### Model building and refinement

Model building for human Ca_v_2.3 used Ca_v_2.2 complex comprising α1, α2δ−1, and β3 subunit (PDB code 7MIY) as the starting model. The structure of Ca_v_2.2 was docked to the Ca_v_2.3 map using UCSF Chimera^45^ and then manually adjusted in COOT^46^. The lipids were manually built to fit into the corresponding densities in the map. The model was refined against the corresponding map by the phenix.real_space_refine program in PHENIX^47^ with secondary structure and geometry restraints. For ΔCH2 structure, the model was manually adjusted and refined in COOT based on the WT structure. Statistics of 3D reconstruction and model refinement can be found in Supplementary Table 1. All structure figures were prepared in UCSF Chimera^45^, ChimeraX^48^ and PyMol^49^.

### 3D variability analysis of CH2_II_-deleted structure (ΔCH2)

The 3D variability analysis was performed in cryoSPARC^32,50^. ΔCH2 particles after Bayesian polish (in total 124,196 particles) were applied for one round 2D classification. 8,161 junk particles were used to generate bad references in 3D ab-initio reconstruction program. 116,035 particles were selected and applied to heterogeneous refinement using 1 good and 2 bad references. The selected 90,380 particles afforded a 3.1 Å reconstruction after non-uniform refinement. The output from non-uniform refinement was applied to 3D variability analysis with three components. Initial results were processed as a simple mode for preview. A set of different subset numbers for intermediate reconstruction were tested and 10 was selected and applied for the final presentation. The first and last frames together with the high-resolution structure of ΔCH2 were used for heterogeneous refinement of the 90,380 particles, resulting in a similar reconstruction as shown in intermediate mode with the portions of particle for bend state (13%) and straight state (14%), respectively.

### Whole-cell voltage-clamp recordings

HEK293T cells (ATCC) were cultured in Dulbecco’s Modified Eagle Medium (DMEM, Gibco) supplemented with 10% (v/v) fetal bovine serum (PAN-Biotech) at 37 °C with 5% CO_2_. For 35 mm culture dish, 0.5 μg cDNA of each subunit, 0.5 μg pIRES2-EGFP (Clontech) plasmid, and 6 μg polyethylenimine (PEI) (Polysciences) were used for transfection. Whole-cell voltage-clamp electrophysiology was performed 36 to 60 hours after transfection at room temperature. Isolated, GFP-positive cells were then selected for whole-cell recordings.

The extracellular solution contained 160 mM TEA-Cl, 1 mM BaCl_2_, 1 mM CaCl_2_, and 10 mM HEPES (pH 7.3). The intracellular solution contained 140 mM CsCl, 10 mM HEPES, and 10 mM EGTA (pH 7.4). Glass pipette electrodes with a resistance of 2-5 MΩ were used. Whole-cell currents were acquired using a MultiClamp 700B Amplifier and a Digidata 1550B digitizer with pCLAMP 10 software (Molecular Devices). Data were collected at a 20 kHz sample rate and filtered at 5 kHz with a low-pass filter. The series resistance was ~5-10 MΩ and was compensated ~80–90%.

To obtain the activation curves of WT and ΔCH2 Ca_v_2.3 channels, cells were held at −100 mV followed by a series of 100-ms voltage steps from −60 mV to +50 mV in 5 mV increments. Data analyses were performed using Clampfit (Molecular Devices) and GraphPad Prism 7 (GraphPad Software). The currents of Ca_v_2.3 were converted into conductance using the following equation:

1 $$g=I/(V-{V}_{{rev}})$$

where *g* represents conductance, *I* for Ca_v_2.3 current, *V* for tested membrane potential, and *V*_*rev*_ for reversal potential. The conductance data were then fitted with the Boltzmann equation below to generate the steady-state activation curve:

2 $$\frac{g}{{g}_{{\max }}}=\frac{1}{1+{e}^{({V}_{{mid}}-V)/k}}$$

Here *g* is the conductance at a test voltage, *g*_*max*_ *is* the maximal conductance of Ca_v_2.3 across tested voltages, *V* is the tested voltage, *V*_*mid*_ is the half-maximal activation voltage, and *k* is the slope factor.

To generate the steady-state inactivation curve, cells were held at −100 mV followed by a series of 1 s voltage steps from −100 mV to +20 mV in 5 mV increments (pre-pulse), followed immediately by a 50 ms test pulse to +10 mV.

The steady-state inactivation curves were then fitted with the Boltzmann equation below:

3 $$\frac{I}{{I}_{{\max }}}=\frac{1}{1+{e}^{({V}_{{mid}}-V)/k}}$$

Here *I* is the current during the test pulse after preconditioning at a certain voltage, *I*_*max*_ is the maximal current of Ca_v_2.3, *V* is the tested pre-pulse voltage, *V*_*mid*_ is the half-maximal inactivation voltage, and *k* is the slope factor.

To test the time-dependent recovery from inactivation, cells were initially held at −100 mV and depolarized to either +10 mV or −40 mV for 300 ms to inactivate channels. A recovery hyperpolarization step to −100 mV was then applied for a variable period (0, 5, 10, 25, 50, 75, 100, 125, 150, 200, 300, 500, 800, and 1000 ms), followed by a 30 ms test pulse to +10 mV. Currents at the test pulse were collected and plotted over the recovery time. The curves showing recovery from inactivation were fitted using the single exponential equation below:

4 $$\frac{I}{{I}_{{\max }}}=1-{e}^{\left(-\frac{t}{\tau }\right)}$$

Here *I* is the current at the test pulse after a certain recovery time, *I*_*max*_ is the current at 1000 ms recovery, *t* is the recovery time, and *τ* is the time constant of recovery from the inactivated state.

### Lipidomic analysis of PIP2

Protein samples were buffer-exchanged to 1 M ammonium acetate, pH 7.0 with 0.0042% GDN, and digested with trypsin overnight at 37 °C. The peptide/lipid mixture was dried using a SpeedVac vacuum concentrator (Thermo Fisher Scientific) and dissolved in 70% mobile phase A (acetonitrile/H2O: 60/40, 10 mM ammonium formate and 0.1% formic acid) and 30% mobile phase B (isopropanol/acetonitrile: 90/10, 10 mM ammonium formate and 0.1% formic acid). The lipids were loaded onto a C8 column (Acclaim PepMap 100, C8, 75 µm × 15 cm, Thermo Scientific) by a Dionex UltiMate 3000 RSLC Nano system coupled to an Eclipse Tribrid mass spectrometer (Thermo Scientific). The lipids were separated with a gradient from 20% to 70% mobile phase B. Typical MS settings were spray voltage of 2.2 kV and heated capillary temperature of 320 °C. For data-dependent acquisition, full MS scans were acquired in the Orbitrap (m/z 300-2000) with a resolution of 120000 in negative ion mode. Fragment spectra were acquired in the Orbitrap with a resolution of 15000 using higher-energy collisional dissociation (HCD) with stepped collision energies (25% and 30%).

The raw LC-MS/MS data were converted to mgf format and processed with LipiDex (v1.1) for phospholipid identification using LipiDex_HCD_Formic and a manually curated glycolipid library^51^. The MS and MS/MS search tolerances were set to 0.01 Th. Phospholipid quantification was also performed using LipiDex. The peak features from raw LC-MS/MS data were firstly extracted using MZmine (v.2.53)^51^ with noise level of 5 × 10^4^. The chromatograms were integrated using local minimum search algorithm with minimum absolute height of 5 × 10^5^ and peak duration range of 0.05-1.50 min. The isotopic peaks were grouped and aligned with m/z tolerance of 0.005 Th/10.0 ppm and retention time tolerance of 0.5 min. The quantified peak features were matched to the identified lipid species with minimum MS2 search dot product of 500 and MS2 search reverse dot product of 700. The quantified lipids were manually examined and normalized for relative quantification.

For phosphoinositides analysis, the LC-MS method was adapted from Ogsio et al. and tailored for identifying co-purified lipids with membrane proteins^52^. Briefly, the protein samples were buffer-exchanged to 1 M ammonium acetate, pH 7.0 with 0.0042% GDN, and digested with trypsin overnight at 37 °C. The peptide/lipid mixture was dried and dissolved in 100% methanol. No phosphoinositide enrichment was performed before LC-MS analysis. The samples were diluted to 50% methanol with water, just before LC-MS analysis. The phosphoinositides were analyzed on a C8 column (PepMap 100, C8, 75 µm × 15 cm, Thermo Fisher Scientific) held at 35 °C using a Dionex UltiMate 3000 RSLC Nano system. A binary buffer system was applied to separate phosphoinositides. Mobile phase A was methanol/water/ethylamine (50/50/0.1) and mobile phase B was isopropanol/ethylamine (100/0.1). The lipids were separated with a liner gradient from 5 % to 90% mobile phase B at a flow rate of 300 nl/min. The LC system was coupled to an Eclipse Tribrid mass spectrometry in negative ion mode (Thermo Scientific). For data-dependent acquisition, full MS scans were acquired in the Orbitrap (m/z 300-1800) with a resolution of 120000. Fragment spectra were acquired in the Orbitrap with a resolution of 15000 using HCD with stepped collision energy (25% and 30%). The raw data was processed manually for phosphoinositide identification and quantification.

### Reporting summary

Further information on research design is available in the Nature Portfolio Reporting Summary linked to this article.

## Supplementary information

Supplementary Information

Description of Additional Supplementary Files

Supplementary Movie 1

Reporting Summary

## Data Availability

The data that support this study are available from the corresponding authors upon reasonable request. The cryo-EM maps have been deposited in the Electron Microscopy Data Bank (EMDB) under the accession codes EMD-28529 (wild-type Ca_v_2.3) and EMD-28530 (ΔCH2 mutant Ca_v_2.3). The coordinates have been deposited in the RCSB Protein Data Bank (PDB) under the accession codes 8EPL (wild-type Ca_v_2.3) and 8EPM (ΔCH2 mutant Ca_v_2.3). The proteins for structural comparison in this study can be found in PDB under the accession code 7MIY (human Ca_v_2.2), and in EMDB under the accession codes EMD-22426 (rabbit Ca_v_1.1) and EMD-23868 (human Ca_v_2.2). The lipidomic raw data have been deposited on Figshare (10.6084/m9.figshare.21502188). The source data underlying Figs. 2a, c, 4b and Supplementary Figs. 1b, 4a, b, 5a, 6a, c are provided as a Source Data file. Source data are provided with this paper.

## Source data

Source Data
