# Supplementary material for: Structures of the R-type human Cav2.3 channel reveal conformational crosstalk of the intracellular segments
Source: Nat Commun. 2022 Nov 30;13:7358. doi: 10.1038/s41467-022-35026-6 (PMC9708679; doi:10.1038/s41467-022-35026-6)
Supplement: Supplementary file 1 — Supplementary Information [file 41467_2022_35026_MOESM1_ESM.pdf]

Supplementary Information

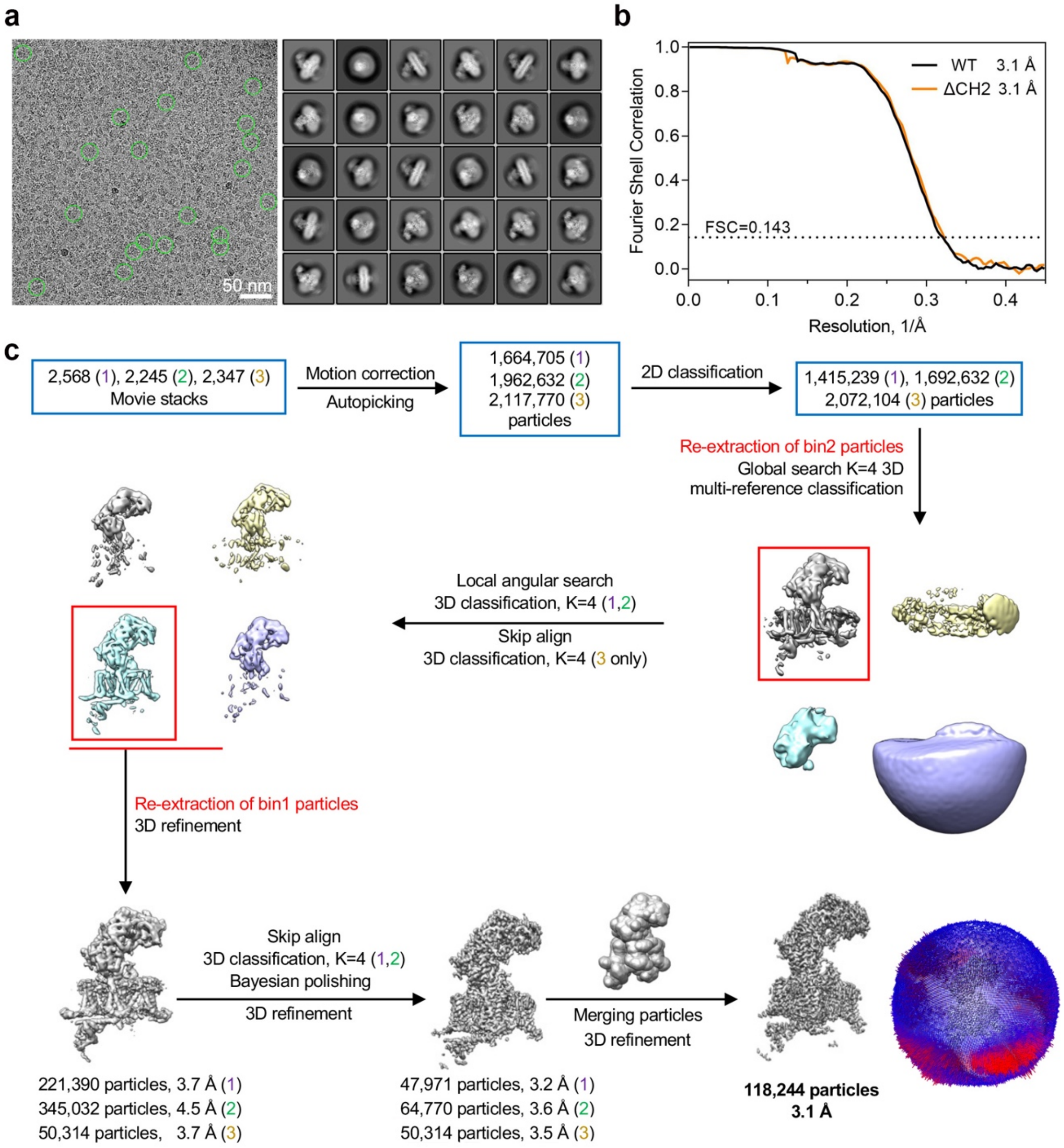

Supplementary Fig. 1 | Structural determination of human Ca$_v$2.3 complex. a A cropped representative cryo-EM micrograph (left) and 30 classes of final 2D classification (right) of wild-type (WT) Ca$_v$2.3 from one batch of experiment. The green circles indicate representative particles in distinct orientations. Box size for the 2D averages is 280 pixels. **b** Gold-standard Fourier Shell Correlation (FSC) curves for the overall 3D reconstructions of WT Ca$_v$2.3 (black line) and ΔCH2 mutant protein (orange line). The FSC curves were calculated using Post-processing program in RELION. Source data are provided as a Source Data file. **c** Cryo-EM data processing of WT Ca$_v$2.3. In total, three batches of data were combined for processing. The 2nd and 3rd datasets were collected

with the addition of SNX-482; however, there was no density discernible for toxin in the 3D EM map. Angular distribution of the final particles is shown alongside the final reconstruction. Each cylinder represents one view and the height of the cylinder is proportional to the number of particles for that view.

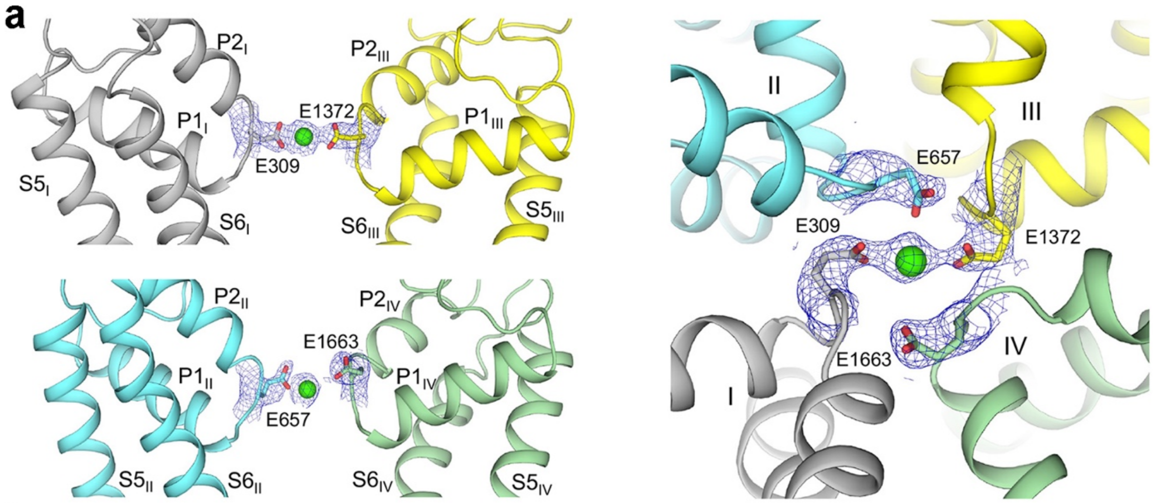

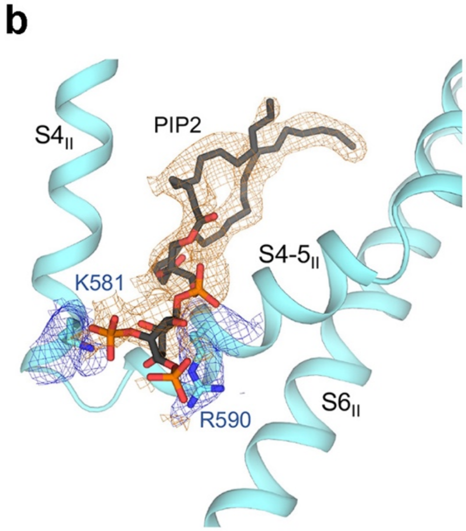

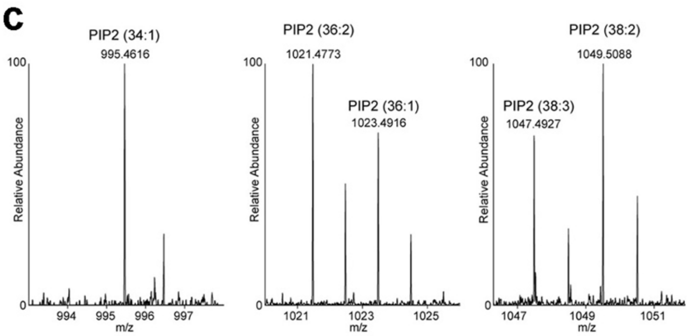

Lipid ID	Observed mass	Theoretical mass	Mass tolerance
PIP2 (34:1)	995.4616	995.4669	5 ppm
PIP2 (36:2)	1021.4773	1021.4825	5 ppm
PIP2 (36:1)	1023.4916	1023.4982	6 ppm
PIP2 (38:3)	1047.4927	1047.4982	5 ppm
PIP2 (38:2)	1049.5088	1049.5138	5 ppm

Supplementary Fig. 2 | Structural determination of selectivity filter and phosphatidylinositol 4,5-bisphosphate (PIP2). a Two side views (left) and an extracellular view (right) of the selectivity filter are shown. The EM densities for the bound Ca^{2+} ion and surrounding residues, shown as blue meshes, are contoured at 6 σ in PyMol. **b** A PIP2 molecule binds at the interface of VSD_{II} and PD. Lys581 and Arg590 may have polar interactions with PIP2. The densities for PIP2 and surrounding residues, shown as orange and blue meshes, are contoured at 3 σ. **c** Mass spectrometry analysis of PIP2 extracted from the WT $Ca_v2.3$. Five PIP2 species, 34:1, 36:2, 36:1, 38:3, and 38:2 PIP2 were detected.

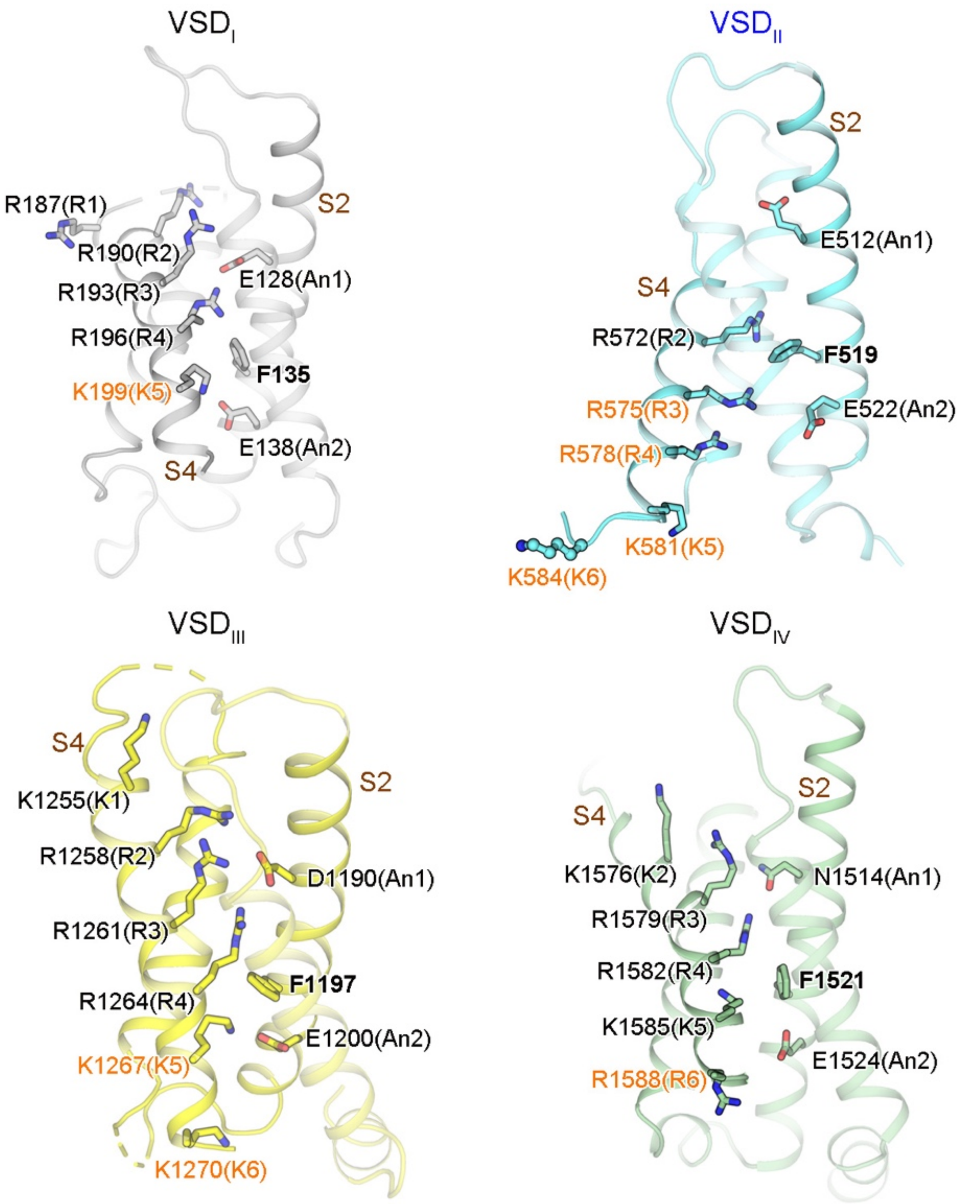

Supplementary Fig. 3 | The four VSDs in WT Ca_v2.3. VSD_{II} is in a down state while the other three VSDs are in the up (depolarized) conformations. The gating charge (GC) residues on S4 and their coordinating residues on S2 are shown as sticks. To make the numbering of the GC residues on each VSD consistent, the GC residue on the first helical turn of S4 is designed as R/K1. The labels for the occluding Phe on S2 are bolded. The GCs above and below the occluding Phe are labeled black and orange, respectively. An1 and An2 represent conserved acidic or polar residues on S2.

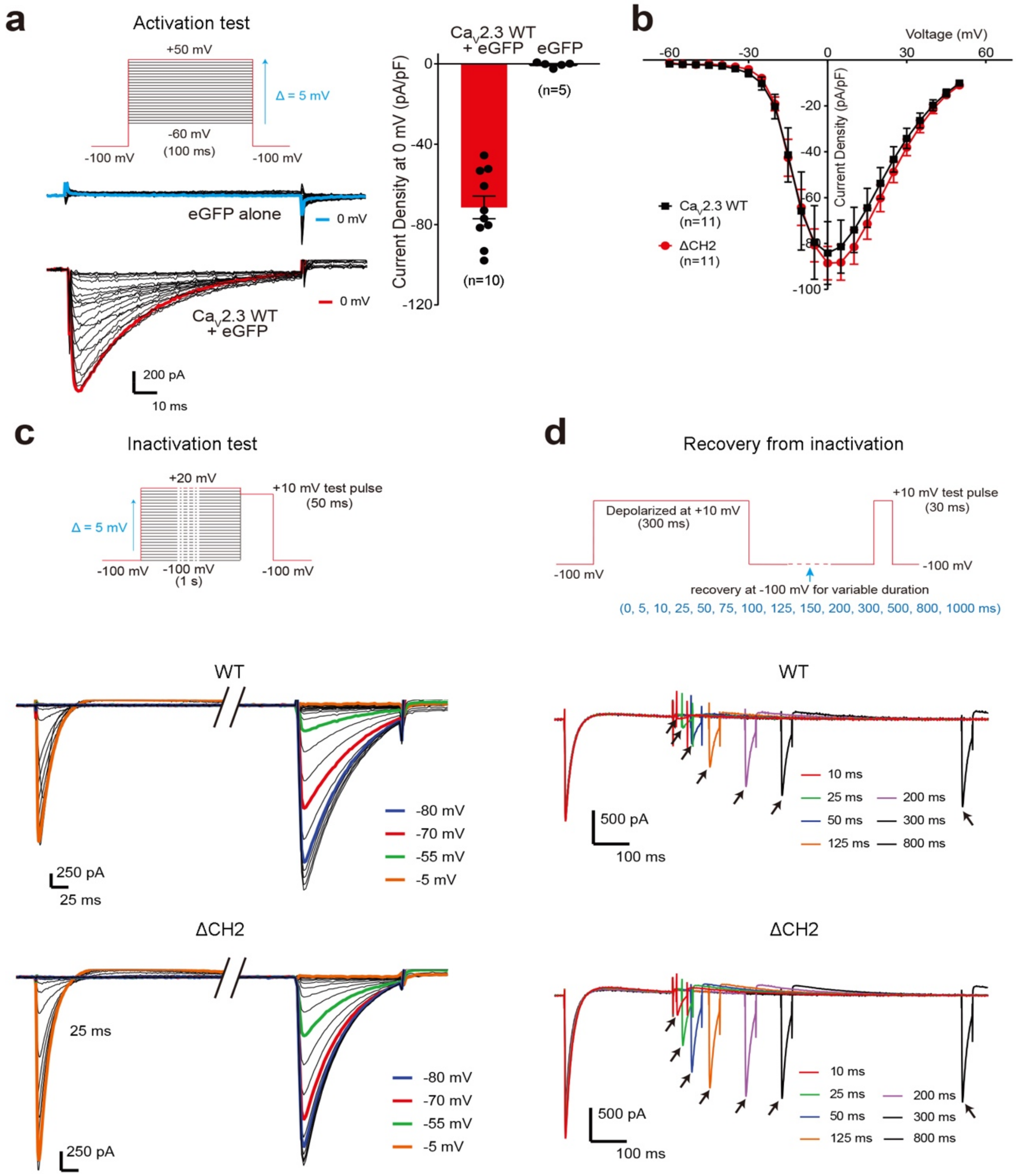

Supplementary Fig. 4 | Electrophysiological studies of WT and ΔCH2 Ca$_v$2.3 channels. a Left: Voltage protocol and the representative currents of the HEK293T cells transfected with plasmids expressing eGFP alone or co-transfected with plasmids expressing the three Ca$_v$2.3 subunits and eGFP. Right: The current densities recorded at 0 mV. Data in this figure are presented as mean ± SEM. The number of tested cells is labeled in parentheses (n=10 for Ca$_v$2.3WT+eGFP; n=5 for eGFP alone). Source data are provided as a Source Data file. **b** The average current-voltage relationship (I-V) curves of the WT and ΔCH2 mutant Ca$_v$2.3 channels are almost identical. n=11 tested cells both for WT and ΔCH2 Ca$_v$2.3. Source data are provided as a Source Data file. **c**

Voltage protocol and the representative currents recorded for plotting the inactivation curves of the WT and the ΔCH2 mutant channels. Current peaks obtained at +10 mV testing pulse were used for calculation. Traces recorded at several indicated inactivation voltages are shown in different colors. **d** Voltage protocol and the representative currents recorded for plotting the inactivation recovery of the WT and the ΔCH2 mutant channels. Protocol and recorded currents with a pre-pulse at +10 mV are shown here as an example. Current peaks (pointed by arrows) obtained at +10 mV testing pulse were used for calculation. Traces recorded at several selected recovery time are shown in different colors.

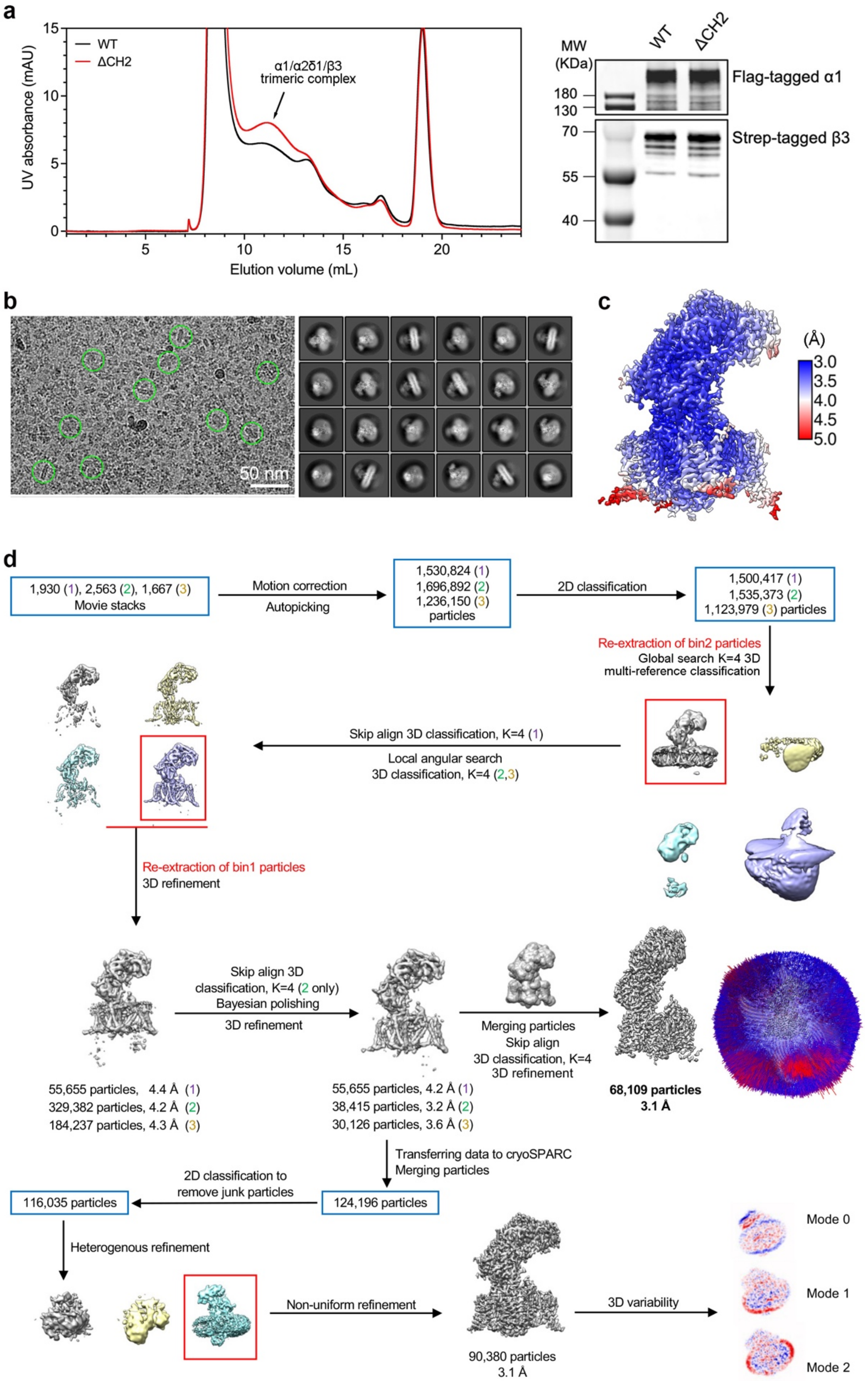
a
UV absorbance (mAU)
Elution volume (mL)
WT
ΔCH2
α1/α2δ1/β3
trimeric complex
MW
(KDa)
180
130
70
55
40
WT
ΔCH2
Flag-tagged α1
Strep-tagged β3
b
50 nm
c
(Å)
3.0
3.5
4.0
4.5
5.0
d
1,930 (1), 2,563 (2), 1,667 (3)
Movie stacks
Motion correction
Autopicking
1,530,824 (1)
1,696,892 (2)
1,236,150 (3)
particles
2D classification
1,500,417 (1)
1,535,373 (2)
1,123,979 (3) particles
Re-extraction of bin2 particles
Global search K=4 3D
multi-reference classification
Skip align 3D classification, K=4 (1)
Local angular search
3D classification, K=4 (2,3)
Re-extraction of bin1 particles
3D refinement
Skip align 3D
classification, K=4 (2 only)
Bayesian polishing
3D refinement
Merging particles
Skip align
3D classification, K=4
3D refinement
55,655 particles, 4.4 Å (1)
329,382 particles, 4.2 Å (2)
184,237 particles, 4.3 Å (3)
55,655 particles, 4.2 Å (1)
38,415 particles, 3.2 Å (2)
30,126 particles, 3.6 Å (3)
68,109 particles
3.1 Å
Transferring data to cryoSPARC
Merging particles
2D classification to
remove junk particles
116,035 particles
124,196 particles
Heterogenous refinement
Non-uniform refinement
3D variability
90,380 particles
3.1 Å
Mode 0
Mode 1
Mode 2

Supplementary Fig. 5 | Structural determination of the ΔCH2 mutant Ca_v2.3. a The protein behavior and the stoichiometry of the α1 and β3 subunits of ΔCH2 are similar to that of WT channel. Left: Size-exclusion chromatography for proteins obtained from 1.5 L of transfected HEK293F cells. Right: Western blotting using the anti-Flag antibody against the α1 subunit and the anti-Strep antibody against the β3 subunit. The eluents after anti-Flag affinity purification were pooled for analysis in two independent experiments with similar results. Source data are provided as a Source Data file. **b** A cropped representative cryo-EM micrograph (left) and 24 classes of final 2D classification (right) of the ΔCH2 mutant Ca_v2.3 from one batch of experiment. The green circles indicate representative particles in distinct orientations. Box size for the 2D averages is 280 pixels. **c** Heat map for the local resolutions of the ΔCH2 Ca_v2.3 EM reconstruction. **d** Cryo-EM data processing workflow. Three batches of data were combined for processing. 3D variability analysis was performed in cryoSPARC. Angular distribution of the final particles is shown alongside the final reconstruction. Each cylinder represents one view and the height of the cylinder is proportional to the number of particles for that view.

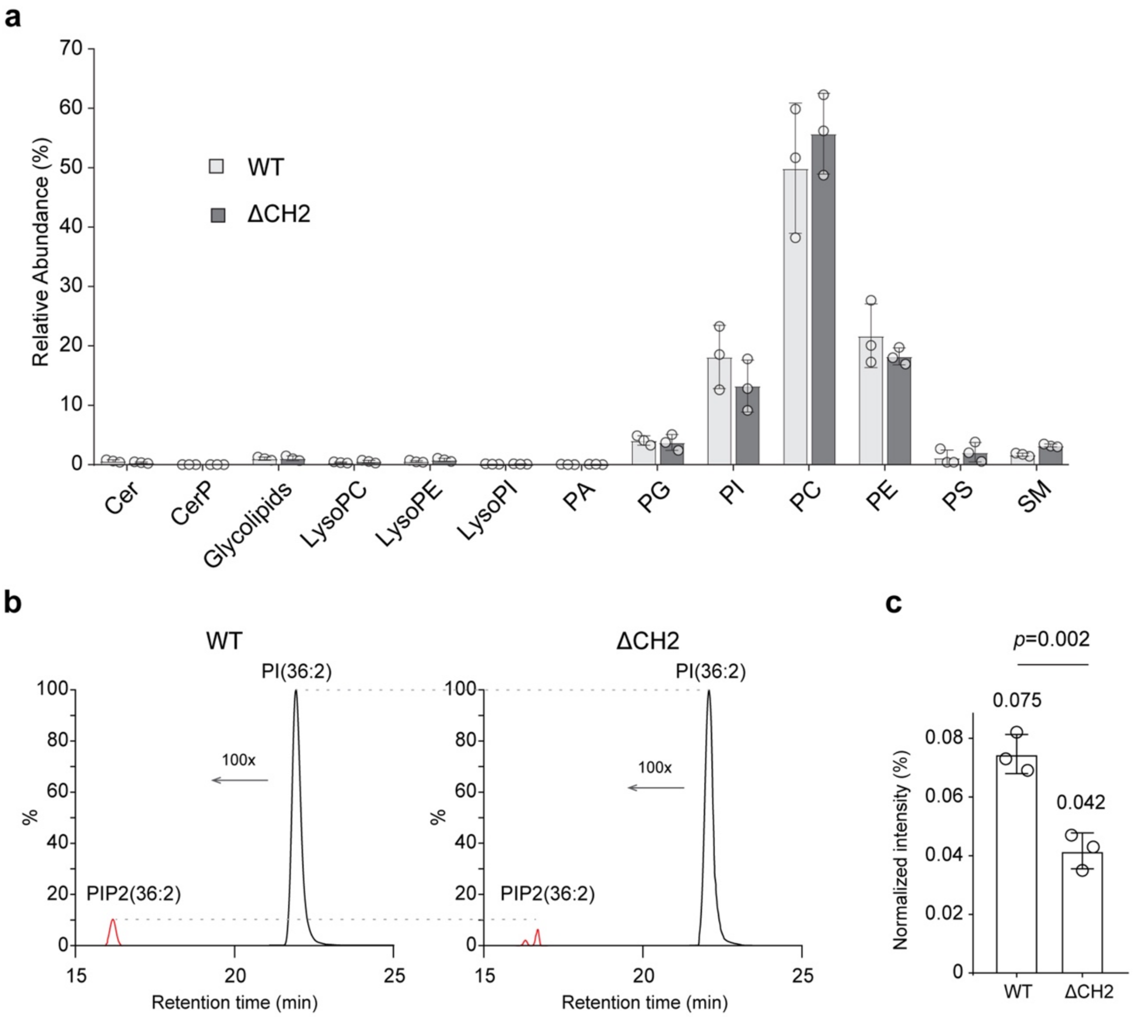

Supplementary Fig. 6 | PIP2 is less abundant in ΔCH2 mutant $Ca_v2.3$. a Lipidomic quantification of the co-purified lipids in WT and ΔCH2 $Ca_v2.3$. Phosphatidylinositol (PI) and phosphatidylethanolamine (PE) are reduced in the ΔCH2 mutant sample. Data in this figure are presented as mean ± SD, n=3 biologically independent experiments. Source data are provided as a Source Data file. **b** Extracted ion chromatograms (EICs) of PIP2 (36:2) and PI (36:2) in WT (left) and ΔCH2 (right) $Ca_v2.3$. The relative abundance of PIP2 (36:2) in each sample was normalized to PI (36:2) abundance. **c** Normalized intensities of PIP2 (36:2) in WT and ΔCH2 protein samples from three replicate experiments. The PIP2 peaks (panel b) were separately integrated. The mean AUC (area under the curve) values were labeled above the bars. Two-tailed unpaired t-test was used for statistical analysis. Source data are provided as a Source Data file.

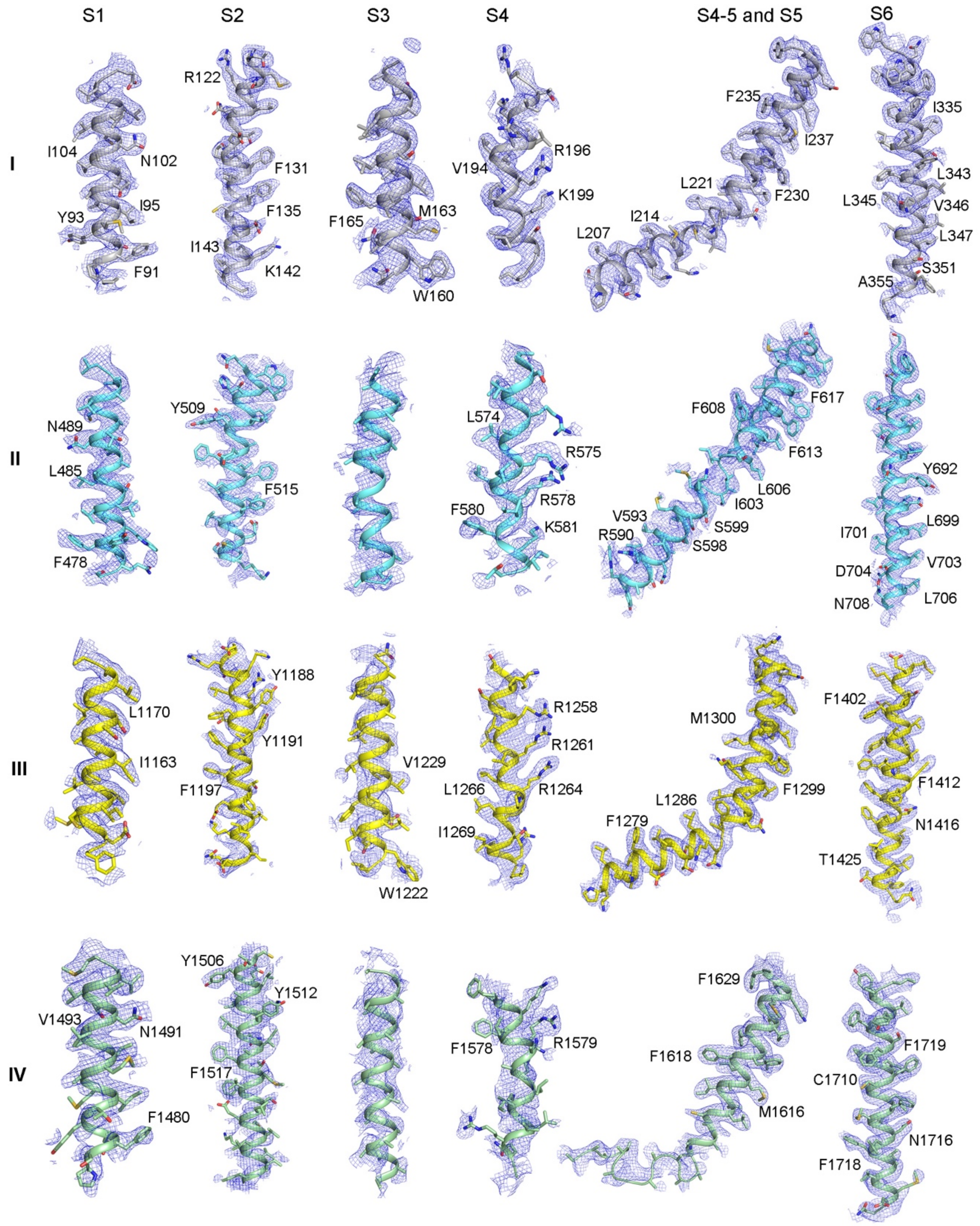

Supplementary Fig. 7 | EM maps for the S1-S6 segments in each repeat of ΔCH2 Ca$_v$2.3. All the densities shown as blue meshes are contoured at 4 σ. Specifically, S3$_{II}$, S3$_{IV}$ and S4-5$_{IV}$ were assigned as poly-Ala because of the low resolutions.

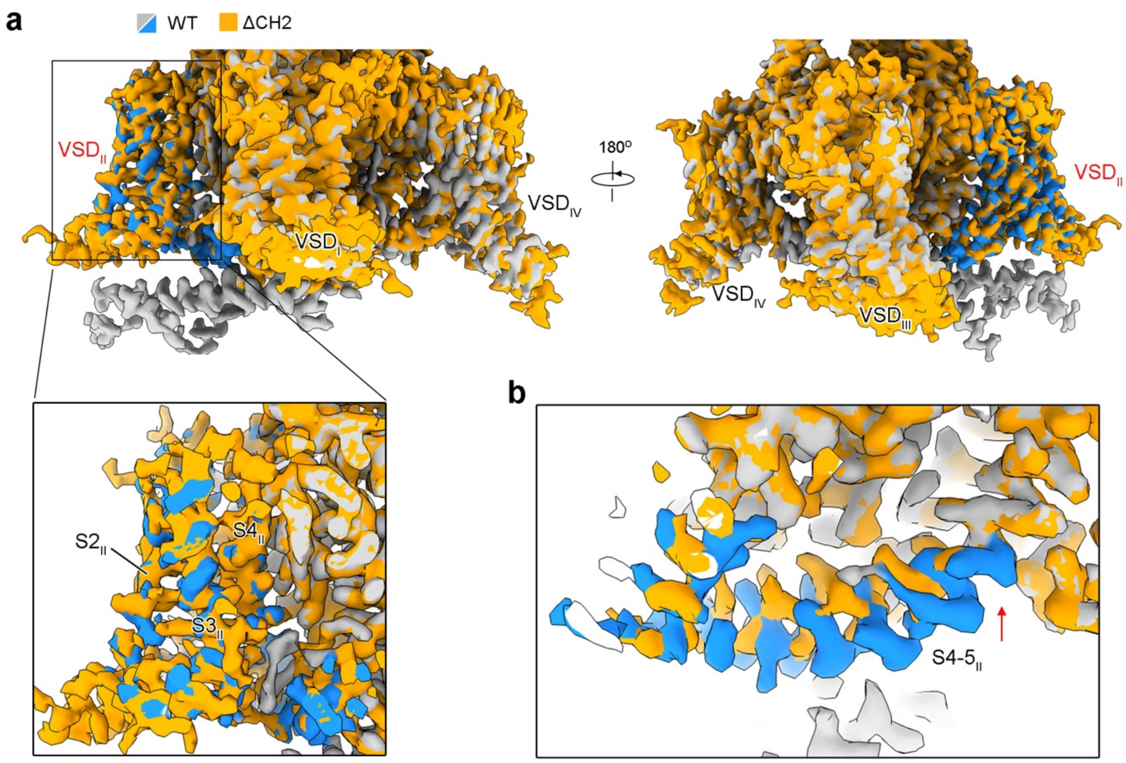

Supplementary Fig. 8 | VSD$_{II}$ in ΔCH2 remains a down state, but the S4-5$_{II}$ displays an evident structural shift. a The overall structures of the α1 subunit of ΔCH2 and WT Ca$_v$2.3 are nearly identical. VSD$_{II}$ and the S4-5$_{II}$ segment in WT reconstruction are highlighted with bright blue. Inset: The densities for VSD$_{II}$ in WT and ΔCH2 Ca$_v$2.3 can be well aligned, supporting that VSD$_{II}$ in ΔCH2 still exhibits down conformation. **b** The upward movement of S4-5$_{II}$ upon deletion of CH2$_{II}$.

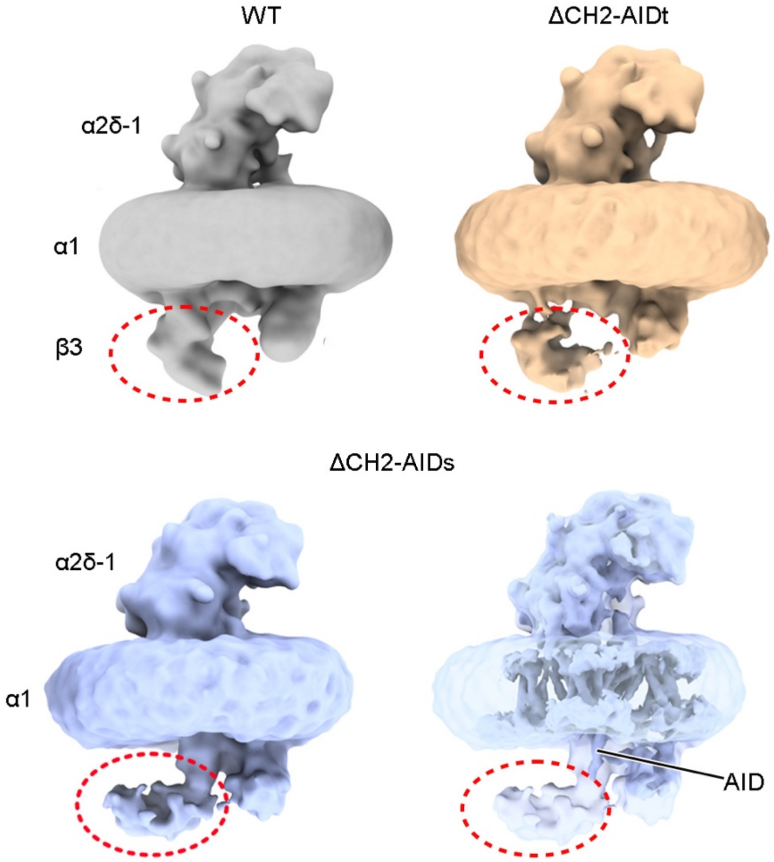

Supplementary Fig. 9 | Two potential conformations of the β3 subunit in ΔCH2 Ca$_v$2.3. To visualize the cytosolic segments, representative reconstructions from 3D variability analysis (3DVA) of ΔCH2 were low pass-filtered to 15 Å. Top: The possible β subunit in ΔCH2-AIDt (wheat, 1st frame of 3DVA) shares similar conformation with WT Ca$_v$2.3 (gray). Bottom: In ΔCH2-AIDs (light blue, 10th frame of 3DVA), blob densities that associated with the straight AID may indicate a distinct position of the β subunit relative to the α1 subunit upon the conformational shift of AID.

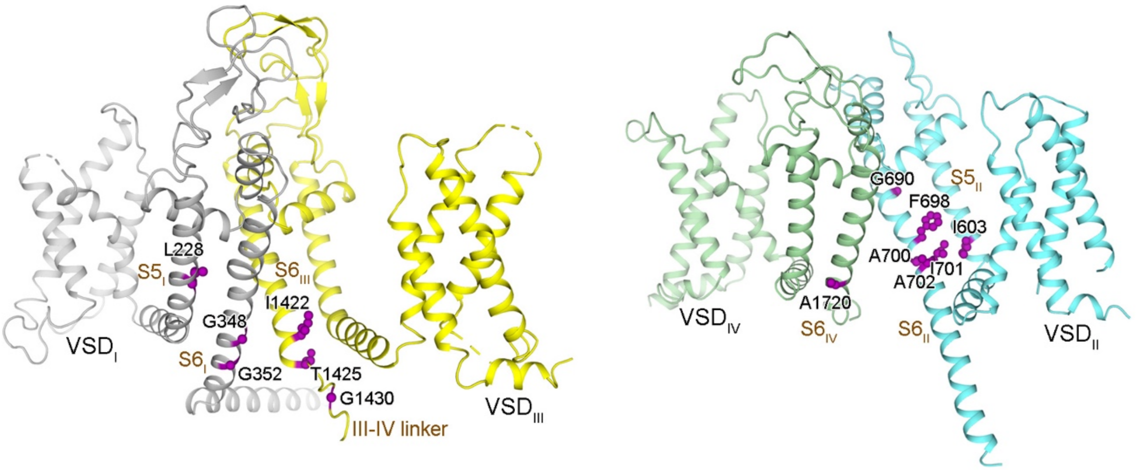

Supplementary Fig. 10 | Structural mapping of the epileptic encephalopathy related mutations identified in Ca_v2.3. The corresponding effects of the mutations can be found in https://www.uniprot.org/uniprotkb/Q15878 and the reference[1]. Two side views of the diagonal repeats are shown. Purple spheres highlight the positions of disease-related residues.

Supplementary Table 1 | Cryo-EM data collection, refinement and validation statistics

	WT $Ca_v2.3$ (EMD-28529) (PDB 8EPL)	$Ca_v2.3$-ΔCH2 (EMD-28530) (PDB 8EPM)
**Data collection and processing**		
Magnification	105,000	105,000
Voltage (kV)	300	300
Electron dose (e-/Å^2)	50	50
Defocus range (μm)	-2.1~-1.9	-2.1~-1.9
Pixel size (Å)	1.114	1.114
Symmetry	C1	C1
Initial particle images (no.)	5,745,107	4,463,866
Final particle images (no.)	118,244	68,109
Map resolution (Å)	3.1	3.1
FSC threshold	0.143	0.143
**Refinement**		
Initial model used (PDB code)	7MIY	WT $Ca_v2.3$
Model resolution (Å)	3.4	3.2
FSC threshold	0.5	0.5
Map sharpening *B* factor (Å^2)	-88	-67
Model composition		
Non-hydrogen atoms	20954	15458
Protein residues	2546	1939
Ligands	25	17
B factors (Å^2)		
Protein	61.38	57.30
Ligand	68.09	96.19
R.m.s deviations		
Bond lengths (Å)	0.003	0.003
Bond angles (°)	0.507	0.531
Validation		
MolProbity score	1.72	1.82
Clashscore	7.22	7.74
Poor rotamers (%)	0.27	0.55
Ramachandran plot		
Favored (%)	95.35	94.08
Allowed (%)	4.57	5.81
Disallowed (%)	0.08	0.10

Supplementary References:

1. Helbig, K.L. et al. De Novo Pathogenic Variants in CACNA1E Cause Developmental and Epileptic Encephalopathy with Contractures, Macrocephaly, and Dyskinesias. *Am J Hum Genet* **103**, 666-678 (2018).
