# Supplementary material for: Structures of the R-type human Cav2.3 channel reveal conformational crosstalk of the intracellular segments
Source: Nat Commun. 2022 Nov 30;13:7358. doi: 10.1038/s41467-022-35026-6 (PMC9708679; doi:10.1038/s41467-022-35026-6)
Supplement: Supplementary file 2 — Description of Additional Supplementary Files [file 41467_2022_35026_MOESM2_ESM.pdf]

Description of Additional Supplementary Files

File Name: Supplementary Movie 1
Description: Dynamic motion of the juxtamembrane domains in ΔCH2 Cav2.3. The movie shows volume series reconstructed from 3D variability analysis in cryoSPARC. The α2δ-1 and α1 subunits are colored with the same scheme as in Fig. 1b.
