# Supplementary material for: Structures of the R-type human Cav2.3 channel reveal conformational crosstalk of the intracellular segments
Source: Nat Commun. 2022 Nov 30;13:7358. doi: 10.1038/s41467-022-35026-6 (PMC9708679; doi:10.1038/s41467-022-35026-6)
Supplement: Supplementary file 4 — Reporting Summary [file 41467_2022_35026_MOESM4_ESM.pdf]

nature portfolio

Corresponding author(s): Nieng Yan

Last updated by author(s): Nov 6, 2022

Reporting Summary

Nature Portfolio wishes to improve the reproducibility of the work that we publish. This form provides structure for consistency and transparency in reporting. For further information on Nature Portfolio policies, see our Editorial Policies and the Editorial Policy Checklist.

Statistics

For all statistical analyses, confirm that the following items are present in the figure legend, table legend, main text, or Methods section.

n/a	Confirmed	
☐	☒	The exact sample size (*n*) for each experimental group/condition, given as a discrete number and unit of measurement
☐	☒	A statement on whether measurements were taken from distinct samples or whether the same sample was measured repeatedly
☐	☒	The statistical test(s) used AND whether they are one- or two-sided<br>*Only common tests should be described solely by name; describe more complex techniques in the Methods section.*
☒	☐	A description of all covariates tested
☒	☐	A description of any assumptions or corrections, such as tests of normality and adjustment for multiple comparisons
☐	☒	A full description of the statistical parameters including central tendency (e.g. means) or other basic estimates (e.g. regression coefficient) AND variation (e.g. standard deviation) or associated estimates of uncertainty (e.g. confidence intervals)
☐	☒	For null hypothesis testing, the test statistic (e.g. *F*, *t*, *r*) with confidence intervals, effect sizes, degrees of freedom and *P* value noted<br>*Give P values as exact values whenever suitable.*
☒	☐	For Bayesian analysis, information on the choice of priors and Markov chain Monte Carlo settings
☒	☐	For hierarchical and complex designs, identification of the appropriate level for tests and full reporting of outcomes
☒	☐	Estimates of effect sizes (e.g. Cohen's *d*, Pearson's *r*), indicating how they were calculated

Our web collection on statistics for biologists contains articles on many of the points above.

Software and code

Policy information about availability of computer code

Data collection	SerialEM 3.8.1, pClamp 10
Data analysis	RELION 3.0, MotionCor2 1.4.0, GCTF 1.18, Phenix 1.20.1 , Coot 0.9.8.1, Pymol 2.4.2, Chimera 1.15.0, ChimeraX_Daily 1.4.0, GraphPad Prism 9.0.2, GraphPad Prism 7.0.0, Clampfit 10, LipiDex v1.1

For manuscripts utilizing custom algorithms or software that are central to the research but not yet described in published literature, software must be made available to editors and reviewers. We strongly encourage code deposition in a community repository (e.g. GitHub). See the Nature Portfolio guidelines for submitting code & software for further information.

Data

Policy information about availability of data

All manuscripts must include a data availability statement. This statement should provide the following information, where applicable:

- Accession codes, unique identifiers, or web links for publicly available datasets
- A description of any restrictions on data availability
- For clinical datasets or third party data, please ensure that the statement adheres to our policy

The cryo-EM maps have been deposited in the Electron Microscopy Data Bank (EMDB) under the accession codes EMD-28529 [https://www.ebi.ac.uk/pdbe/entry/emdb/EMD-28529] (wild-type Cav2.3) and EMD-28530 [https://www.ebi.ac.uk/pdbe/entry/emdb/EMD-28530] (ΔCH2 mutant Cav2.3). The coordinates have been deposited in the RCSB Protein Data Bank (PDB) under the accession codes 8EPL [http://doi.org/10.2210/pdb8EPL/pdb] (wild-type Cav2.3) and 8EPM [http://doi.org/10.2210/pdb8EPM/pdb] (ΔCH2 mutant Cav2.3). The proteins for structural comparison in this study can be found in PDB under the accession code 7MIY

[http://doi.org/10.2210/pdb7MIY/pdb] (human Cav2.2), and in EMDB under the accession codes EMD-22426 [https://www.ebi.ac.uk/pdbe/entry/emdb/EMD-22426] (rabbit Cav1.1) and EMD-23868 [https://www.ebi.ac.uk/pdbe/entry/emdb/EMD-23868] (human Cav2.2). The lipidomic raw data have been deposited on Figshare (https://doi.org/10.6084/m9.figshare.21502188). All data reported by this study are publicly available as of the date of publication.
The source data underlying Figs. 2a, 2c, 4b and Supplementary Figs. 1b, 4a, 4b, 5a, 6a, 6c are provided as a Source Data file.

Human research participants

Policy information about studies involving human research participants and Sex and Gender in Research.

Reporting on sex and gender	N/A
Population characteristics	N/A
Recruitment	N/A
Ethics oversight	N/A

Note that full information on the approval of the study protocol must also be provided in the manuscript.

Field-specific reporting

Please select the one below that is the best fit for your research. If you are not sure, read the appropriate sections before making your selection.

- [x] Life sciences
- [ ] Behavioural & social sciences
- [ ] Ecological, evolutionary & environmental sciences

For a reference copy of the document with all sections, see nature.com/documents/nr-reporting-summary-flat.pdf

Life sciences study design

All studies must disclose on these points even when the disclosure is negative.

Sample size	In our electrophysiology recording, more than 10 cells were tested for WT and mutant. The number is selected based on our previous experience and what people generally do in the electrophysiology field. This size is sufficient for performing the statistical test. The sample size was not applied to the structural and lipidomic analysis, because these experiments studied the cryo-EM structures or the molecular weight of the lipids extracted from WT and mutant proteins.
Data exclusions	No data were excluded from the analyses.
Replication	Replication was not applied to the structural analysis. For electrophysiology recording and lipidomic analysis, all data have been successfully repeated with at least two batches of samples and all results were similar.
Randomization	The randomization is not relevant to our study. Our experiments only studied cryo-EM structures, electrophysiological properties and lipid abundance of WT and mutant proteins.
Blinding	All the constructs were recorded and analyzed blindly to avoid bias.

Reporting for specific materials, systems and methods

We require information from authors about some types of materials, experimental systems and methods used in many studies. Here, indicate whether each material, system or method listed is relevant to your study. If you are not sure if a list item applies to your research, read the appropriate section before selecting a response.

Materials & experimental systems

n/a	Involved in the study
[ ]	[x] Antibodies
[ ]	[x] Eukaryotic cell lines
[x]	[ ] Palaeontology and archaeology
[x]	[ ] Animals and other organisms
[x]	[ ] Clinical data
[x]	[ ] Dual use research of concern

Methods

n/a	Involved in the study
[x]	[ ] ChIP-seq
[x]	[ ] Flow cytometry
[x]	[ ] MRI-based neuroimaging

Antibodies

Antibodies used	Monoclonal ANTI-FLAG® M2 antibody (Sigma-Aldrich, F3165-1MG); Strep Tag II monoclonal antibody (Invitrogen, MA5-37747, clone 1810CT579.47.56.10); IRDye® 800CW goat anti-mouse IgG secondary antibody (Li-COR, 926-32210)
Validation	https://www.sigmaaldrich.com/US/en/product/sigma/f3165<br>https://www.thermofisher.com/antibody/product/Strep-Tag-II-Antibody-clone-1810CT579-47-56-10-Monoclonal/MA5-37747<br>https://www.licor.com/bio/reagents/irdye-800cw-goat-anti-mouse-igg-secondary-antibody

Eukaryotic cell lines

Policy information about cell lines and Sex and Gender in Research

Cell line source(s)	HEK293F (Invitrogen), HEK293T(ATCC)
Authentication	No further authentication was performed for commercially available cell lines.
Mycoplasma contamination	Not tested for mycoplasma contamination.
Commonly misidentified lines<br>(See ICLAC register)	No commonly misidentified cell lines were used.
